# The neurons that restore walking after paralysis

**DOI:** 10.1038/s41586-022-05385-7

**Published:** 2022-11-09

**Authors:** Claudia Kathe, Michael A. Skinnider, Thomas H. Hutson, Nicola Regazzi, Matthieu Gautier, Robin Demesmaeker, Salif Komi, Steven Ceto, Nicholas D. James, Newton Cho, Laetitia Baud, Katia Galan, Kaya J. E. Matson, Andreas Rowald, Kyungjin Kim, Ruijia Wang, Karen Minassian, John O. Prior, Leonie Asboth, Quentin Barraud, Stéphanie P. Lacour, Ariel J. Levine, Fabien Wagner, Jocelyne Bloch, Jordan W. Squair, Grégoire Courtine

**Affiliations:** 1 Defitech Center for Interventional Neurotherapies (NeuroRestore), EPFL/CHUV/UNIL, Lausanne, Switzerland; 2 NeuroX Institute and Brain Mind Institute, School of Life Sciences, Swiss Federal Institute of Technology (EPFL), Lausanne, Switzerland; 3 Department of Clinical Neuroscience, Lausanne University Hospital (CHUV) and University of Lausanne (UNIL), Lausanne, Switzerland; 4 Michael Smith Laboratories, University of British Columbia, Vancouver, British Columbia Canada; 5 Spinal Circuits and Plasticity Unit, National Institute of Neurological Disorders and Stroke, Bethesda, MD USA; 6 Bertarelli Foundation Chair in Neuroprosthetic Technology, Laboratory for Soft Bioelectronic Interfaces, Institute of Electrical and Microengineering, Institute of Bioengineering, NeuroX Institute, EPFL, Geneva, Switzerland; 7 Department of Nuclear Medicine and Molecular Imaging, Lausanne University Hospital (CHUV) and University of Lausanne (UNIL), Lausanne, Switzerland; 8 Center for Medical Physics and Biomedical Engineering, Medical University of Vienna, Vienna, Austria

**Keywords:** Spinal cord injury, Genetics of the nervous system

## Abstract

A spinal cord injury interrupts pathways from the brain and brainstem that project to the lumbar spinal cord, leading to paralysis. Here we show that spatiotemporal epidural electrical stimulation (EES) of the lumbar spinal cord^1–3^ applied during neurorehabilitation^4,5^ (EES^REHAB^) restored walking in nine individuals with chronic spinal cord injury. This recovery involved a reduction in neuronal activity in the lumbar spinal cord of humans during walking. We hypothesized that this unexpected reduction reflects activity-dependent selection of specific neuronal subpopulations that become essential for a patient to walk after spinal cord injury. To identify these putative neurons, we modelled the technological and therapeutic features underlying EES^REHAB^ in mice. We applied single-nucleus RNA sequencing^6–9^ and spatial transcriptomics^10,11^ to the spinal cords of these mice to chart a spatially resolved molecular atlas of recovery from paralysis. We then employed cell type^12,13^ and spatial prioritization to identify the neurons involved in the recovery of walking. A single population of excitatory interneurons nested within intermediate laminae emerged. Although these neurons are not required for walking before spinal cord injury, we demonstrate that they are essential for the recovery of walking with EES following spinal cord injury. Augmenting the activity of these neurons phenocopied the recovery of walking enabled by EES^REHAB^, whereas ablating them prevented the recovery of walking that occurs spontaneously after moderate spinal cord injury. We thus identified a recovery-organizing neuronal subpopulation that is necessary and sufficient to regain walking after paralysis. Moreover, our methodology establishes a framework for using molecular cartography to identify the neurons that produce complex behaviours.

## Abstract (web summary)

Transcriptomic analysis following epidural electrical stimulation of the lumbar spinal cord during neurorehabilitation in mice identifies a population of neurons that orchestrates the restoration of walking following paralysis.

## Main

The neurons that orchestrate walking reside in the lumbar spinal cord^14^. To walk, the brain broadcasts commands through descending pathways that cascade from the brainstem to activate these neurons^15,16^. A severe spinal cord injury (SCI) scatters this exquisitely organized communication system^15^. Whereas the neurons located in the lumbar spinal cord are not directly damaged by the injury, the depletion of essential supraspinal commands renders them nonfunctional^4^. The consequence is permanent paralysis.

Isolated case studies have reported that EES can immediately reactivate nonfunctional neurons in the lumbar spinal cord^17,18^, enabling people with paralysis to walk^1,18–20^. Application of EES during neurorehabilitation (EES^REHAB^) further improved recovery of walking, even when the stimulation was turned off^1,21^.

The biological principles through which EES^REHAB^ engages and remodels the lumbar spinal cord to restore walking remain unknown. Here, we hypothesized that EES^REHAB^ must engage and remodel essential yet unidentified neurons in the spinal cord that become necessary for walking after paralysis.

## EES^REHAB^ remodels the human spinal cord

We first tested whether EES^REHAB^ can restore walking across a large population of individuals with SCI, and whether this recovery involves remodelling of the lumbar spinal cord.

**Fig. 1:**
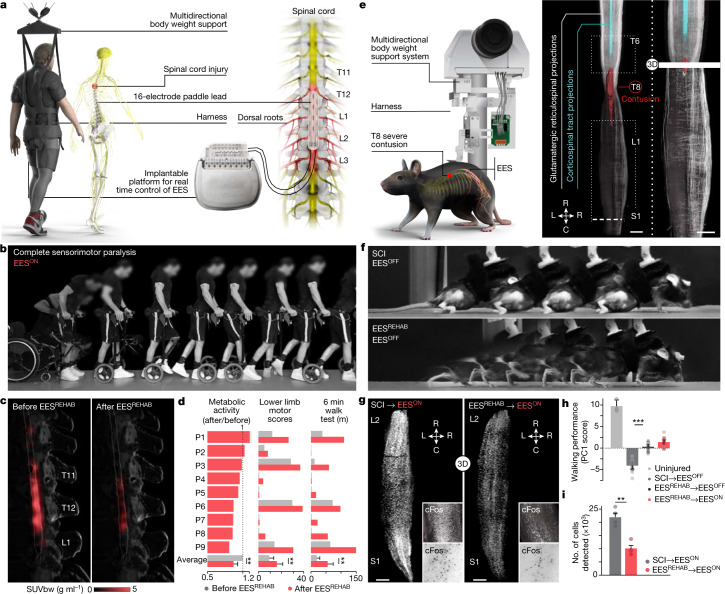
EES^REHAB^ remodels the spinal cord of humans and mice. **a**, Body weight support system enabling overground walking and wireless implantable pulse generator operating in closed loop, connected to a paddle lead targeting the dorsal roots that innervate lumbosacral segments. **b**, Chronophotography showing the transitioning from sitting to walking in a representative participant. **c**, ^18^FDG-PET projected onto a personalized model of the spinal cord elaborated from high-resolution MRI (participant ID DM002), showing the metabolic activity of the spinal cord—expressed as standardized uptake value (SUV_bw_)—in response to walking before and after EES^REHAB^. **d**, Bar plots reporting the relative change in normalized FDG-PET metabolic activity during walking before and after EES^REHAB^, the lower limb motor scores, and the distance covered during the 6-min walk test (*n* = 9; metabolic activity mixed-effects model: *t* = −3.2, *P* = 0.002; lower limb motor scores, paired samples two-tailed *t*-test: *t* = 3.7, *P* = 0.0063; distance covered, paired samples two-tailed *t*-test: *t* = 3.5; *P* = 0.0076). **e**, Left, body weight support system enabling overground walking in mice, with implantable electrodes to deliver EES. Right, spinal cord visualization of projections from neurons in the motor cortex and glutamatergic (vGluT2^ON^) neurons in the reticular formation, traced with AAV5-CAG-COMET-GFP and AAV5-CAG-DIO-COMET-tdTomato, respectively. Scale bars, 1 mm. **f**, Chronophotography of representative mice with SCI only (SCI, EES^OFF^) or SCI with EES^REHAB^ (EES^REHAB^, EES^OFF^). **g**, Lumbar spinal cord expression of cFos following walking with EES^ON^ following SCI or SCI with EES^REHAB^. Scale bars, 500 μm. **h**, Walking performance of uninjured mice (*n* = 3), mice with SCI (*n* = 10), and mice with SCI and EES^REHAB^ tested with EES^OFF^ (*n* = 10) or EES^ON^ (*n* = 10) (one-way ANOVA; Tukey’s honest significant difference for SCI versus EES^REHAB^→EES^OFF^: *P* = 3.3 × 10^–11^). **i**, The number of neurons expressing cFos(cFos^ON^) (mice with SCI with EES^ON^, *n* = 4; mice with EES^REHAB^ and EES^ON^, *n* = 4; independent samples two-tailed *t*-test: *t* = –5.7; *P* = 0.001). **h**,**i**, Bars show mean ± s.e.m. with individual points overlaid. *P < 0.05, **P < 0.01, ***P < 0.001. Source data

Nine individuals were enroled in the first-in-human clinical trial ‘Stimulation Movement Overground’ (STIMO) (clinicaltrials.gov: NCT02936453; Supplementary Information), which aimed to establish the safety and feasibility of EES^REHAB^ to improve the recovery of walking in people with chronic SCI. EES^REHAB^ combines a surgically implanted neurostimulator interfaced to a multi-electrode paddle lead that enables closed-loop control of biomimetic EES protocols^1,2,18^, and overground neurorehabilitation supported in a multidirectional robotic support system^1,22^ (Fig. 1a and Supplementary Table 1). The first six participants presented with severe or complete motor paralysis, but all of them had retained some degree of sensation in the legs. The other three participants presented with complete sensorimotor paralysis. The first six participants were implanted with a repurposed paddle lead originally developed to treat neuropathic pain^1^ and the last three were implanted with a newly designed, purpose-built paddle lead that aimed to target the ensemble of thoracic, lumbar and sacral dorsal roots involved in the production of walking^18^.

**Extended Data Fig. 1:**
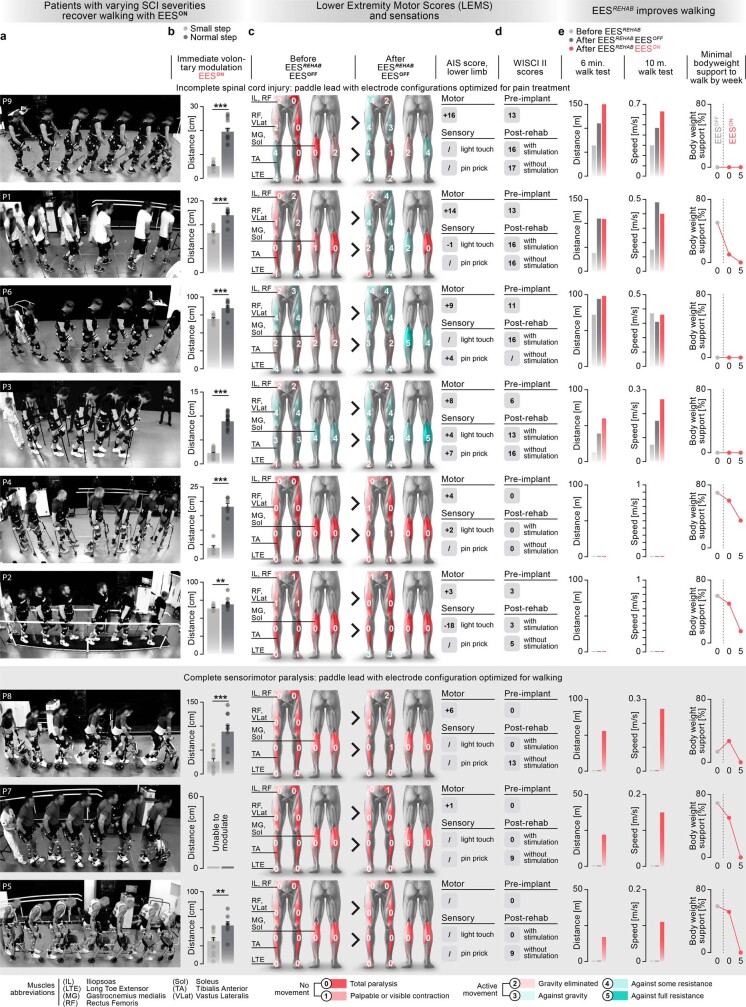
EES^REHAB^ restores walking in nine humans with chronic SCI. **a**, Chronophotography of nine human participants with chronic SCI who are walking independently with EES^ON^ and the help of crutches or a front-wheel walker at the end of the neurorehabilitation period. **b**, Bar plots reporting the volitional modulation of the step for each participant during EES^ON^ (paired samples two-tailed t-tests: P9: *n* = 13 small and 12 large steps, t = 10.6, *P* = 2.3 × 10^–10^; P1: *n* = 13 small and 9 large steps, t = 6.4, *P *= 3.1 × 10^–6^; P6: *n* = 13 small and 10 large steps, t = 4.8, *P* = 9.9 × 10^–6^; P3: *n* = 32 small and 24 large steps, t = 23.0, P < 10^–15^; P4: *n* = 10 small and 7 large steps, t = 12.1, P < 10^–15^; P2: *n* = 15 small and 15 large steps, t = 3.0, *P* = 0.006; P8: *n* = 15 small and 10 large steps, t = 5.3, *P* = 2.0 × 10^–5^; P5: *n* = 12 small and 8 large steps, t = 3.0, *P* = 0.0073). **c**, Body schemes report the lower extremity motor score (LEMS) measured before EES^REHAB^ and after EES^REHAB^. Relative changes in lower limb motor and sensory scores after EES^REHAB^ are also reported. **d**, WISCIII score measured before EES^REHAB^ and after EES^REHAB^ with EES^OFF^ and EES^ON^. **e**, Bar plots reporting the outcome of the clinically standardized six-minute walk test and 10-meters walk test before EES^REHAB^, and after EES^REHAB^ with EES^OFF^ and EES^ON^. Participants used a front-wheel walker for stability, but did not receive any external assistance. Two participants could not walk without assistance at the hip or at least 10 to 30% of body weight support for stability. Consequently, they did not perform these evaluations. Line plots report the evolution of the required amount of body weight support (% of bodyweight) necessary to enable each participant to walk independently over the period of EES^REHAB^. Bar plots show the mean with individual data points overlaid. Error bars show the standard error of the mean. *, *P* < 0.05; **, *P* < 0.01; ***, *P* < 0.001. Source data

Biomimetic EES protocols immediately enabled all nine participants to improve or regain the ability to walk while supported in the robotic interface (Extended Data Fig. 1a). Moreover, the participants, including two with complete sensorimotor paralysis, could exert volitional control over the amplitude of their steps when EES was turned on (Extended Data Fig. 1b).

After the initial phase of configuration, the participants underwent EES^REHAB^ for five months, which consisted of standing, walking and performing various exercises with EES^ON^ four to five times per week. Weight-bearing capacities improved considerably over time (Extended Data Fig. 1e), which enabled the participants to walk outdoors with EES^ON^ and an assistive device for stability (Supplementary Video 1). Participants who exhibited residual function before EES^REHAB^ displayed a pronounced increase in lower limb motor scores that restored walking even in the absence of EES in four participants (Fig. 1d and Extended Data Fig. 1c–e). These results support the primary and secondary endpoints of the clinical trial.

The sustained recovery of walking suggested that EES^REHAB^ remodels the spinal cord. We speculated that this remodelling must be reflected in the activity of neurons during walking. To address this possibility, we quantified the metabolic activity of the spinal cord in response to walking before and after EES^REHAB^ using positron emission tomography (PET) of ^18^F-fluorodeoxyglucose (FDG) uptake (^18^FDG-PET). Walking elicited pronounced activity within lumbar segments (Fig. 1c). Unexpectedly, EES^REHAB^ led to a decrease in this activity (Fig. 1c,d).

This reduction in the neuronal activity in the lumbar spinal cord supported the hypothesis that EES^REHAB^ steers activity-dependent selection of specific neuronal subpopulations that become essential for walking after paralysis.

## EES^REHAB^ remodels the mouse spinal cord

We reasoned that identifying the neuronal subpopulations selected during the recovery of walking with EES^REHAB^ would require a preclinical model in which genetically defined neuronal subpopulations could be catalogued, dissected and manipulated. Therefore, we established a translational framework in mice to replicate the key technological and therapeutic features of EES^REHAB^ in humans.

**Extended Data Fig. 2:**
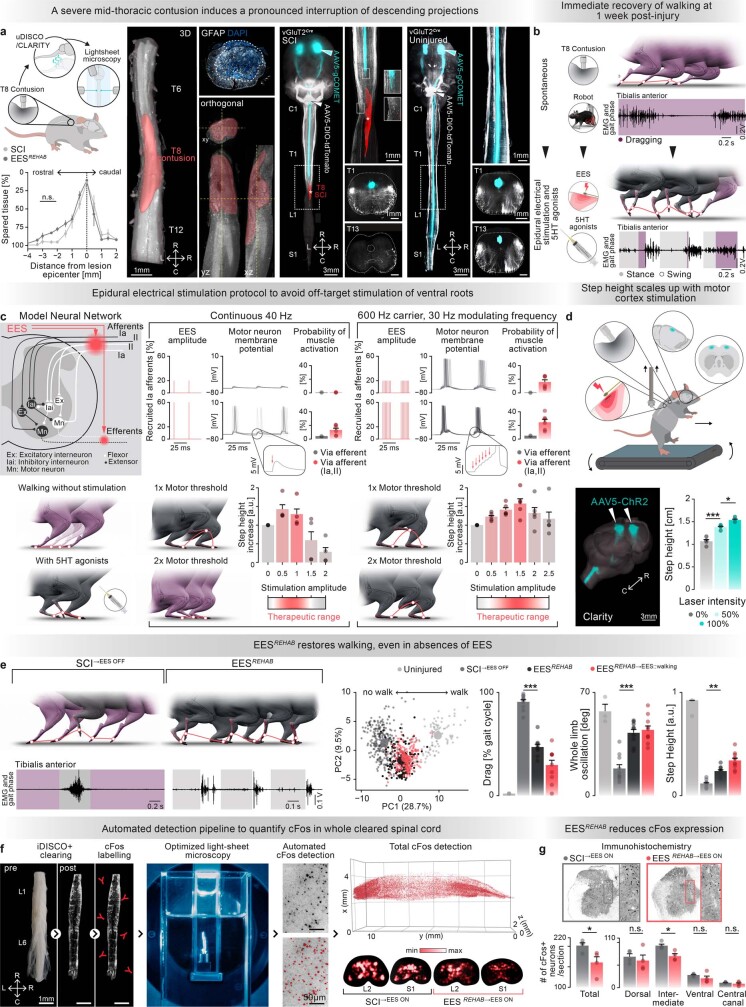
Mouse model replicating the key technological and therapeutic features of EES^REHAB^ in humans. **a**, Three-dimensional visualization and quantification of the contusion SCI. Photographs show multiple 3D views of a contused spinal cord that has been cleared using uDISCO^71^. The extent of spinal cord damage was quantified from coronal sections immunolabeled against glial fibrillary acidic protein (GFAP). The relative amount of spared tissues was quantified at the lesion epicentre and at regular intervals from the lesion epicentre in the rostral and caudal directions, as reported in the line plots. Sparing was consistent across groups (mean = 10.6%, *n* = 5 mice per group; independent samples two-tailed t-test on lesion epicenter, t = 0.5, *P* = 0.61). The impact of the contusion SCI on descending pathways compared to an uninjured spinal cord was visualized in whole brain-spinal cord preparations cleared with CLARITY. Projections from neurons located in the leg region of the motor cortex (AAV5-CAG-COMET-GFP) and from GluT2^ON^ neurons located in the reticular formation (AAV5-CAGDIO-COMET-tdTomato) were labeled with virus infusions, as indicated in the photograph. Insets show the complete interruption of corticospinal fibers and partial preservation of reticulospinal fibers. **b**, Adaptations of the technological features of EES^REHAB^ in mice, including a new robotic body weight support interface with 1g accuracy and a pair of electrodes attached to L2 and S1 spinal segments to deliver EES. Kinematic recordings of whole-body movements were mapped onto a 3D model of the mouse including bones and skin contours to generate realistic visualization of leg movements^40^. The line shows the trajectory of the lower limb endpoint (toe) while circles indicate the maximum step height. Representative leg movements are shown concomitantly to the electromyographic (EMG) activity of the tibialis anterior, recorded from chronically implanted bipolar electrodes into the muscle. At one week, EES was insufficient to reactivate the spinal cord to a level that enabled walking in mice. Consequently, a small bolus of agonists to 5H1A/7 (8-OH-DPAT, 0.05-0.2 mg/kg) and 5HT2A receptors (quipazine 0.2-0.3 mg/kg) was administered to augment the response of the spinal cord^4^. The combination of EES and 5-HT agonists enabled all the mice to walk overground as early as one week after the contusion SCI. 5-HT agonists were used to enable sustained walking during EES^REHAB^, but the amount of drug was progressively decreased over the course of the recovery, and eventually suppressed. Final testing was performed without 5-HT agonists. **c**, We noticed that compared to rats and humans, EES was not as effective to enable walking in mice with SCI. Inspection of muscle responses to EES revealed that the stimulation recruited motor nerves (efferents) at relatively low amplitudes. This off-target stimulation of the ventral roots is due to the relatively small size of the mouse spinal cord. We previously showed that high-frequency bursts are effective to maximize the activation of large-diameter afferent fibers targeted by EES^38^. To adapt this stimulation protocol to mice, we elaborated a computational model of the mouse spinal cord^37^. The model includes a spiking neural network model of muscle spindle feedback circuits for a pair of antagonistic muscles. Response in motor neurons are shown for two amplitudes of EES that correspond to the recruitment of 20% and 60% of the entire afferent population. Simulations suggested that, compared to conventional protocols (40 Hz), high-frequency burst stimulation (carrier frequency 600 Hz, modulating frequency 30 Hz) leads to a summation of synaptic events into motor neurons, which augment the probability to activate muscles via the recruitment of afferents compared to efferents. To confirm these findings experimentally, we compared conventional stimulation protocols to high-frequency burst stimulation during stepping on a treadmill in mice with contusion SCI. Reconstructed leg movements including foot trajectories are shown for each experimental condition at two amplitudes of EES. High frequency burst stimulation increased the therapeutic window through which stimulation could be delivered. Concretely, step height scaled linearly up to 1.5× motor threshold using burst stimulation protocols, and stimulation could be delivered effectively up to 2.5× motor threshold. In contrast, conventional stimulation at 40 Hz facilitated stepping around motor threshold, but additional increase in amplitude that would be necessary to promote robust stepping led to tonic activation of leg muscle activation and thus cessation of stepping. **d**, To model the volitional control of stepping observed in humans during EES^ON^, we manipulated cortical activity with optogenetics. We expressed channelrhodopsin in the neurons of the leg motor cortex using targeted injections of AAV5-hSyn-ChR2 (*n* = 4 mice). Mice with contusion SCI were recorded during stepping on a treadmill with EES^ON^ (no 5-HT agonists). Photostimulation of the motor cortex induced a significant increase of the step height that scaled up with laser intensity, as shown in the bar plots (*n* = 4 mice per group; statistics indicate Tukey HSD tests following one-way ANOVA, *P* = 0.0003, *P* = 0.025, respectively). **e**, Leg movements and muscle activity during overground walking without any intervention or support recorded at four weeks after a contusion SCI in a mouse that did not undergo EES^REHAB^ and in a mouse that underwent EES^REHAB^. Locomotor performance was quantified using principal component analysis applied to 80 gait parameters calculated from kinematic recordings (Supplementary Table 2). In this denoised space, each dot represents a gait cycle (*n* = 20 per mouse, *n* = 9 mice per group). Larger dots represent the mean of each experimental group. The first principal component (PC1) distinguished gaits from mice with SCI that did not undergo EES^REHAB^ from mice that underwent EES^REHAB^. Locomotor performances were thus quantified as the scores on PC1 (reported in Fig. 1g). Analysis of factor loadings on PC1 revealed that the percentage of paw dragging, the extent of whole-limb oscillation (virtual limb connecting the hip to the toe) and step height were the parameters that showed the highest correlation with PC1. Bars report the mean values of these gait parameters (*n* = 9 mice per group; statistics indicate Tukey HSD tests following oneway ANOVA, *P* = 2.1 × 10^–9^, *P* = 7.4 × 10^–6^, *P* = 0.0055, respectively). Data from mice that underwent EES^REHAB^ are shown during EES^OFF^ and EES^ON^. **f**, Photographs show whole mouse spinal cords before and after processing with iDISCO+^69,70^, during which the spinal cords underwent immunolabeling of cFos followed by clearing. The spinal cords were imaged with the mesoSPIM lightsheet microscope^84^. Representative microscopy images show a raw coronal optical slice of the cFos labelling in the spinal cord and after application of automated 3D nuclear spot detection. Images were then reconstructed to visualize the entire lumbar spinal cord. **g**, 3D cFos quantifications were confirmed using immunohistochemistry and labelling for cFos on coronal sections of the lumbar spinal cord, as illustrated in the representative photographs of spinal cord sections from mice with SCI and mice that followed EES^REHAB^. The bar plot reports the mean number of cFos labeled cells per section in the spinal grey matter across the whole section, in Lamina I–III (dorsal), in Lamina IV–VI (intermediate), in Lamina VII–IX (ventral) and in Lamina X (central canal) (*n* = 4 mice per group; independent samples two-tailed t-test, t = –2.7, *P* = 0.042; t = 0.60, *P* = 0.57; t = 3.7, *P* = 0.010; t = 1.2, *P* = 0.27; t = 1.0, *P* = 0.35, respectively). Bar plots show the mean with individual data points overlaid. Error bars show the standard error of the mean. *, *P* < 0.05; **, *P* < 0.01; ***, *P* < 0.001. Source data

Mice received a severe mid-thoracic contusion that replicates the most common pathophysiology of human SCIs (Fig. 1e and Extended Data Fig. 2a). Virus-mediated neuronal tract-tracing combined with CLARITY-optimized light-sheet microscopy^23^ revealed the complete interruption of corticospinal tract fibres and pronounced depletion of glutamatergic reticulospinal fibres below the injury (Fig. 1e and Extended Data Fig. 2a). This SCI induced permanent paralysis (Fig. 1f and Extended Data Fig. 2b).

We then leveraged previous designs^24^ to construct a new robotic interface optimized to support the small body weight of mice (Fig. 1e). We also engineered EES protocols that avoid off-target recruitment of ventral roots caused by the small size of the mouse spinal cord (Extended Data Fig. 2c). These protocols increased the range of stimulation amplitudes over which stepping could be improved (Extended Data Fig. 2c). When EES was turned on, the mice immediately regained the ability to walk while supported in the robotic interface (Extended Data Fig. 2b,c).

To model the voluntary modulation of walking observed in humans when EES is turned on, we manipulated the activity of motor cortex projection neurons using optogenetics^25–28^ (Extended Data Fig. 2d). We previously showed that after a contusion SCI, glutamatergic neurons located in the ventral gigantocellular (vGi neurons) relay commands from motor cortex neurons to the lumbar spinal cord to modulate leg muscle activity^28^. Here, we found that activating motor cortex neurons during EES induced an immediate increase in step height that scaled up with laser intensity (Extended Data Fig. 2d).

We incorporated all these developments into an EES^REHAB^ protocol that restored walking in all the tested mice (Fig. 1h, Extended Data Fig. 2e and Supplementary Video 2). This recovery persisted when EES was turned off (Extended Data Fig. 2e).

We then asked whether EES^REHAB^ induces a reduction in neuronal activity in the lumbar spinal cord of mice during walking, as observed in humans (Fig. 1c,d). To answer this question, we performed whole-spinal-cord labelling of cFos—a marker of transcription induced by neuronal activity^29^ (Extended Data Fig. 2f). CLARITY-optimized light-sheet microscopy enabled the automated detection of cFos in response to walking throughout the lumbar spinal cord. We found a pronounced reduction in the number of cFos^ON^ neurons in mice that had undergone EES^REHAB^ compared with mice that did not undergo EES^REHAB^ (Fig 1g,i and Extended Data Fig. 2f,g).

Together, these results demonstrate that our framework recapitulates the key technological and therapeutic features of EES^REHAB^ observed in humans, thus providing the necessary experimental conditions to identify the neuronal subpopulations selected by EES^REHAB^ to restore walking after paralysis.

## Molecular cartography of walking

We anticipated that identifying the neuronal subpopulations that were engaged and remodelled with EES^REHAB^ would require an atlas that catalogues the molecular responses of each neuronal subpopulation across the key therapeutic features of EES^REHAB^. To generate this atlas, we leveraged high-throughput technologies that allowed us to interrogate the spinal cord at single-cell resolution.

**Fig. 2:**
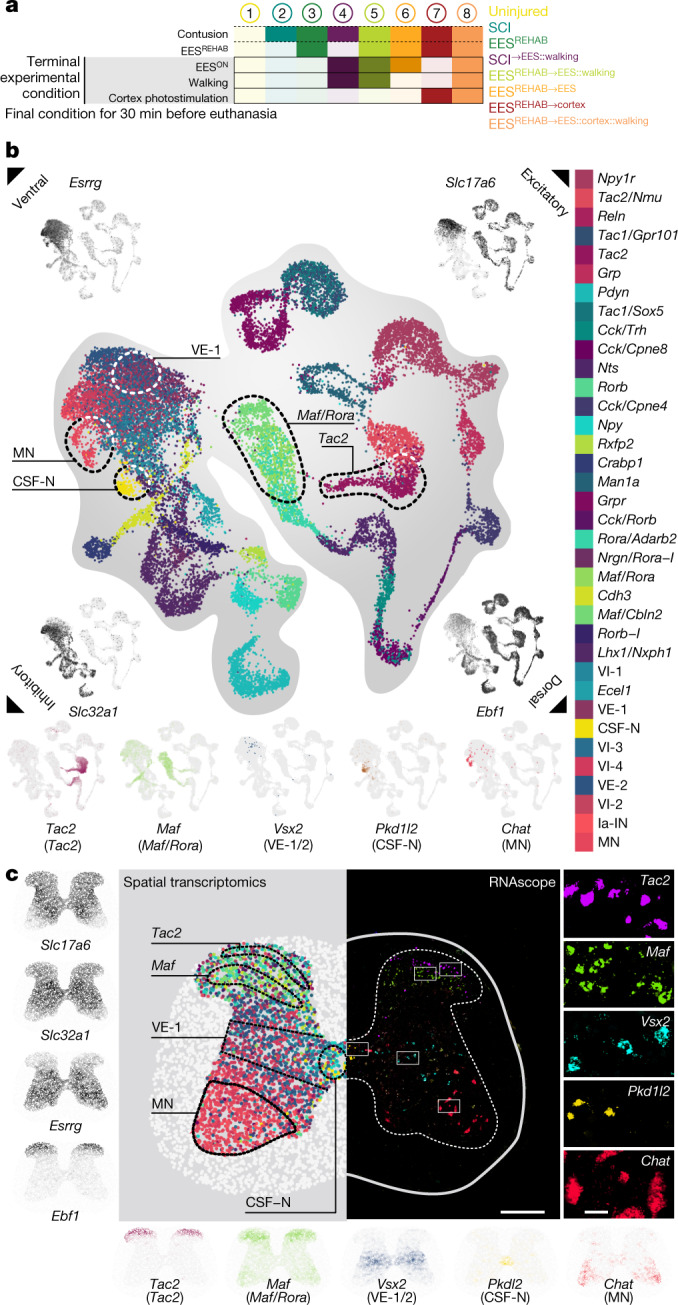
Molecular cartography of EES^REHAB^. **a**, Overview of the eight experimental conditions capturing the key therapeutic features of EES^REHAB^. A detailed description is provided in  Methods, ‘Experimental conditions’. **b**, Uniform manifold approximation and projection (UMAP) visualization of 20,990 nuclei revealing 36 neuron subpopulations. Five dorsal and ventral populations are highlighted on the basis of their marker genes. In each corner, an UMAP visualization coloured by the expression of classical marker genes reveals the cardinal organization of neuronal subpopulations across dorsal–ventral and excitatory–inhibitory axes^51^. MN, motor neuron; VI, ventral-inhibitory; VE, ventral-excitatory; CSF-N, cerebrospinal-fluid contacting neurons; Ia-IN, Ia inhibitory interneurons; Rora-I, inhibitory neurons expressing *Rora*; Rorb-I, inhibitory neurons expressing *Rorb*. **c**, Left, visualization of 22,127 barcodes registered to a common coordinate framework highlighting the expression of classical excitatory–inhibitory and ventral–dorsal marker genes. Second from left, the localization of all 36 neuron subpopulations, with each spatial barcode coloured according to cellular identity. Five classical dorsal and ventral populations are highlighted, with the spatial expression of their marker genes shown below the image. Right, RNAscope analysis, confirming the spatial location of these five neuronal subpopulations.

**Extended Data Fig. 3:**
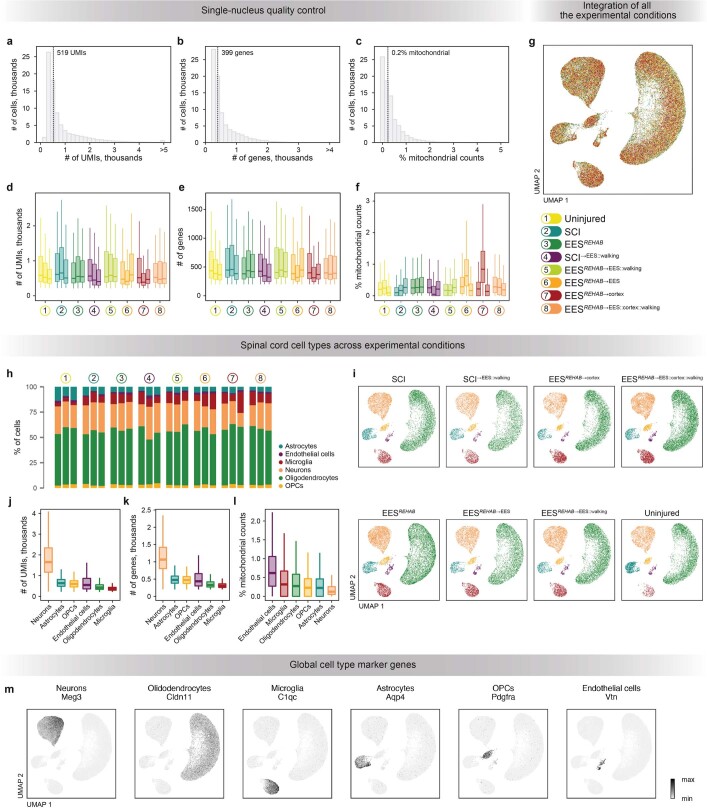
Single-nucleus RNA-sequencing of the mouse lumbar spinal cord. **a–f**, Quality control statistics for 82,093 nuclei from the mouse lumbar spinal cord. **a**, Number of unique molecular identifiers (UMIs) per nucleus. Inset text shows the median number of genes detected. **b**, Number of genes detected per nucleus. Inset text shows the median number of genes detected. **c**, Proportion of mitochondrial counts per nucleus. Inset text shows the median proportion of mitochondrial counts. **d**, As in **a**, but shown for individual libraries from each experimental condition. **e**, As in **b**, but shown for individual libraries from each experimental condition. **f**, As in **c**, but shown for individual libraries from each experimental condition. **d–f**, *n* = 3 mice per condition. **g**, UMAP visualization of 82,093 nuclei colored by experimental condition, revealing consistent recovery of all major transcriptional states in each experimental condition. **h**, Proportions of each major cell type of the lumbar spinal cord recovered in individual libraries from each experimental condition. **i**, UMAP visualizations of nuclei from each experimental condition (*n* = 3 mice per condition). **j**, Number of UMIs quantified per nucleus in each major cell type of the mouse spinal cord. **k**, Number of genes detected per nucleus in each major cell type of the mouse spinal cord. **l**, Proportion of mitochondrial counts per nucleus in each major cell type of the mouse spinal cord. **j–l**, *n* = 3 mice per condition. **m**, Expression of key marker genes for the six major cell types of the lumbar spinal cord. Box plots show the median (horizontal line), interquartile range (hinges) and smallest and largest values no more than 1.5 times the interquartile range (whiskers), and the error bars show the standard deviation.

**Extended Data Fig. 4:**
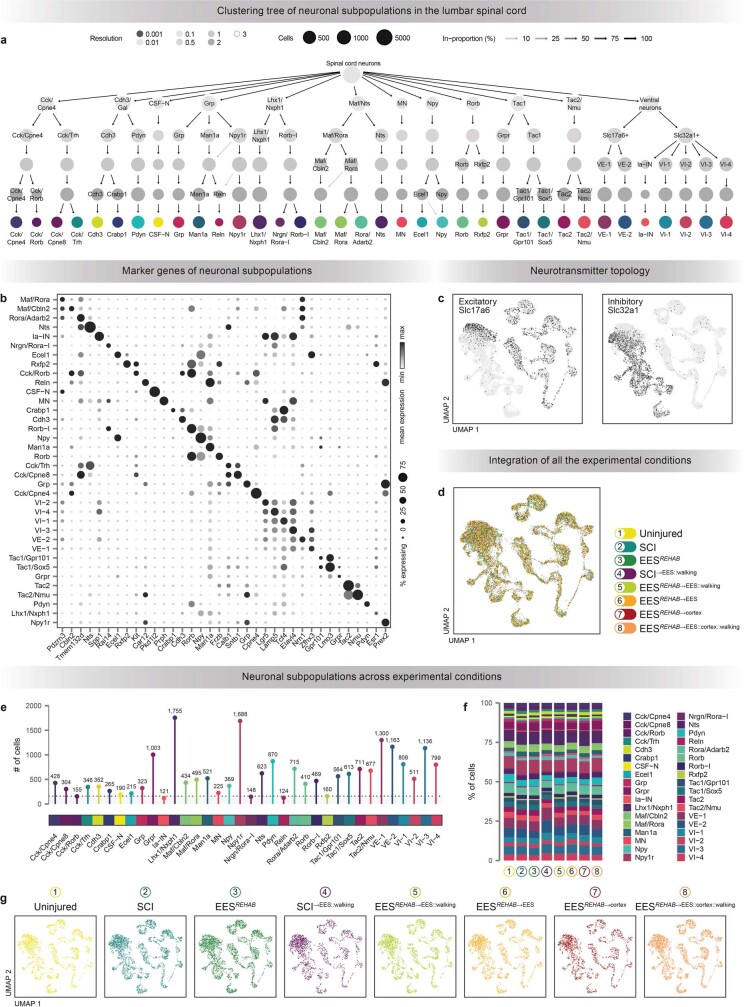
A single-nucleus atlas of neuronal subpopulations in the mouse lumbar spinal cord. **a**, Clustering tree 52 of neurons in the mouse lumbar spinal cord, revealing a hierarchical taxonomy of transcriptionally defined neuron subpopulations over six clustering resolutions. **b**, Dot plot showing expression of a single marker gene per cell type for the 37 transcriptionally defined neuron subpopulations of the mouse lumbar spinal cord. **c**, Expression of key neurotransmitters for excitatory (Slc17a6) and inhibitory (Slc31a1) genes demonstrating a logical topography of neurons in the spinal cord. **d**, UMAP visualization of 20,990 neuronal nuclei colored by experimental condition, revealing consistent recovery of all major neuron transcriptional states in each experimental condition. **e**, Number of nuclei from each transcriptionally defined neuron subpopulation identified across all experimental conditions. **f**, Proportions of each neuronal subpopulation of the lumbar spinal cord recovered in each of eight experimental conditions. **g**, UMAP visualizations of neurons from each experimental condition (*n* = 3 mice per condition).

We first used single-nucleus RNA sequencing^6–9^ (snRNA-seq) to profile the lumbar spinal cord of mice. We devised a progression of eight experimental conditions (Fig. 2a) that captured the key therapeutic features of EES^REHAB^, including terminal conditions executed for 30 min immediately prior to euthanasia (denoted with ^→final_condition^). We obtained high-quality transcriptomes from 82,093 nuclei that were evenly represented across 24 mice from all eight conditions (Extended Data Fig. 3a–g). Unsupervised clustering^30^ identified all of the major cell types of the mouse spinal cord (Extended Data Fig. 3h–m). We then subjected the 20,990 neurons to a second round of clustering, which identified 36 subpopulations of neurons expressing classical marker genes (Fig. 2b and Supplementary Video 3). Our taxonomy recapitulated the known hierarchical organization of neuronal subpopulations in the spinal cord^9,12,31–33^ (Extended Data Fig. 4a–g).

We detected a clear segregation of spinal cord neurons based on the expression of classical dorsal and ventral marker genes (Fig. 2b). This dichotomy compelled us to map our single-cell cartography onto the cytoarchitecture of the spinal cord. We therefore leveraged spatial transcriptomics^10,11^ to resolve the spatial distribution of gene expression in the lumbar spinal cord across key features of EES^REHAB^ (Fig. 2c).

**Extended Data Fig. 5:**
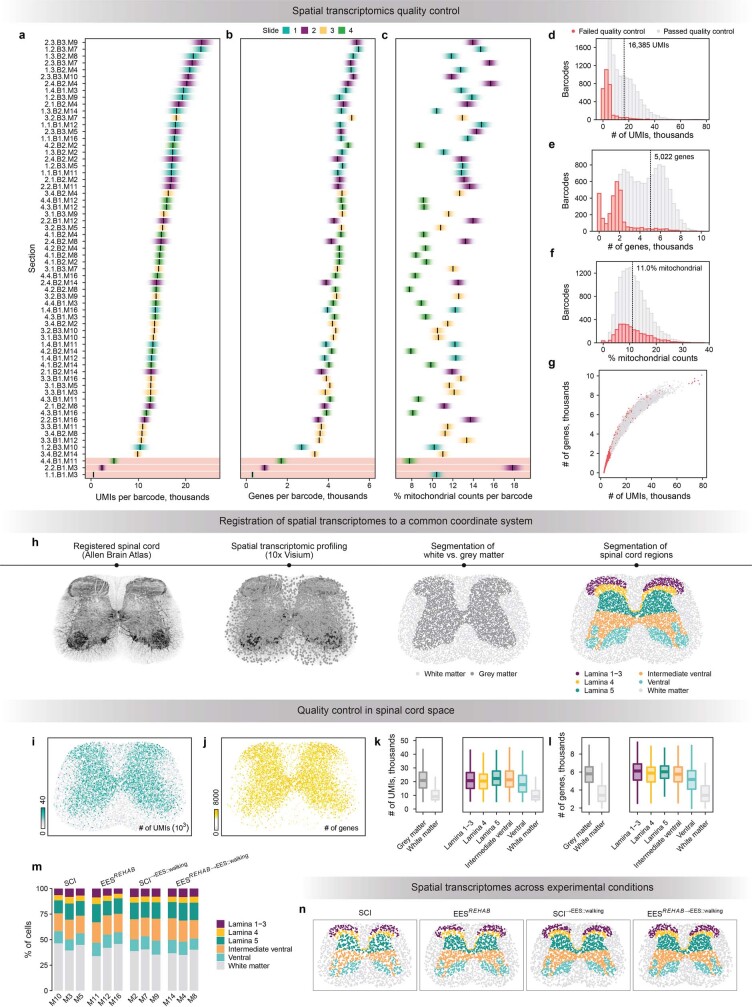
A spatial transcriptomic atlas of the mouse lumbar spinal cord. **a–c**, Quality control statistics for 64 sections from the lumbar spinal cord profiled by spatial transcriptomics (**a**, mean number of UMIs per barcode; **b**, mean number of genes detected per barcode; **c**, mean proportion of mitochondrial counts per barcode). Shaded areas show the standard deviation. Sections are colored by the slide on which each section was sequenced. Sections highlighted in red failed quality control and were removed prior to further analysis. **d–f**, Overall distributions of quality control statistics aggregated over all sections (**d**, number of UMIs per barcode; **e**, number of genes detected per barcode; **f**, proportion of mitochondrial counts per barcode), shown separately for barcodes that passed or failed quality control, respectively. **g**, Relationship between the number of UMIs and number of genes detected per spatial barcode, with barcodes that failed quality control highlighted. **h**, Schematic overview of the registration procedure used to establish a cohesive atlas of spatial transcriptomes, with all 61 spinal cords registered to a common coordinate system. **i, j**, Quality control statistics visualized within the common coordinate system of the mouse lumbar spinal cord (**i**, number of UMIs per barcode; **j**, number of genes detected per barcode). **k–l**, Quality control statistics shown separately for each region of the lumbar spinal cord identified by registration to the Allen Brain Atlas (**k**, number of UMIs per barcode; **l**, number of genes detected per barcode; n = 61 sections from 12 mice). **m**, Proportion of barcodes from each spinal cord region recovered in individual replicates from each of four experimental conditions. **n**, Visualization of barcodes from each of four experimental conditions within the common coordinate system of the mouse lumbar spinal cord. Box plots show the median (horizontal line), interquartile range (hinges) and smallest and largest values no more than 1.5 times the interquartile range (whiskers), and the error bars show the standard deviation.

We sequenced 61 high-quality sections to a median depth of 16,384 UMIs per barcode (Extended Data Fig. 5a–n). To enable comparisons across experimental conditions, we registered all 22,127 barcodes within these 61 sections to a common coordinate system^34^ (Fig. 2c and Extended Data Fig. 5h).

**Extended Data Fig. 6:**
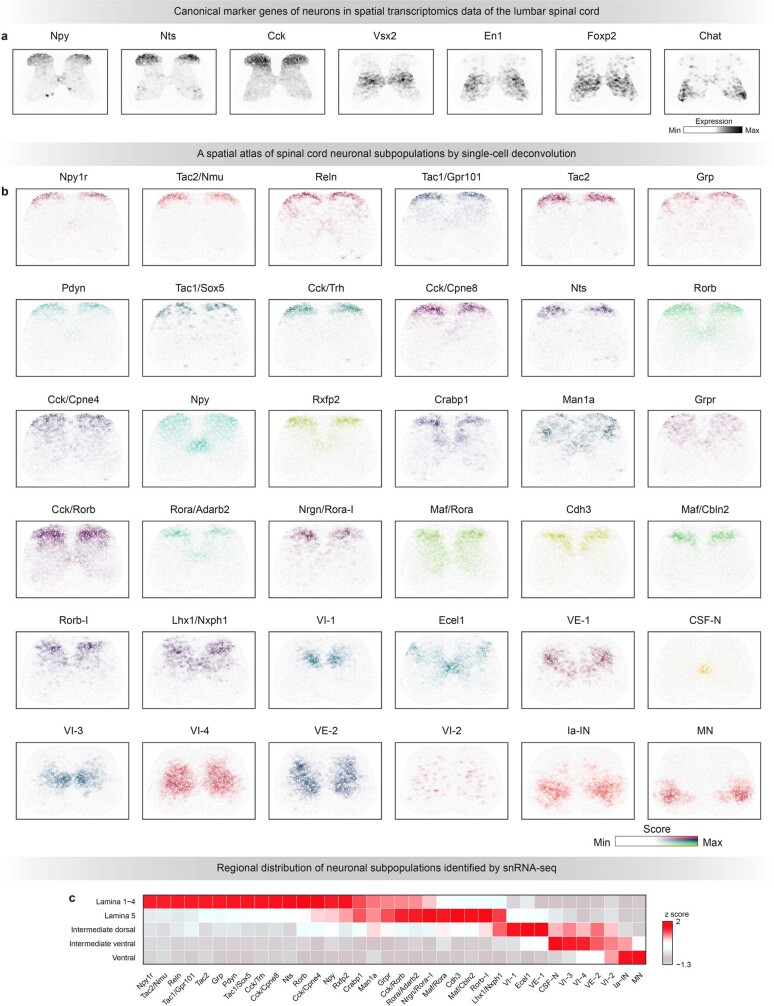
Integration of single-nucleus and spatial transcriptomes. **a**, Spatial expression of key marker genes for canonical neuronal subpopulations of the mouse lumbar spinal cord. **b**, Deconvolution of spatial barcodes using snRNA-seq data to establish a spatial atlas of neuronal subpopulations. Panels show the score assigned by robust cell type decomposition (RCTD) to each of the 36 neuronal subpopulations for each barcode in the spatial transcriptomics dataset. **c**, Mean RCTD scores assigned for each of the 36 neuronal subpopulations to barcodes within five regions of the spinal cord.

**Extended Data Fig. 7:**
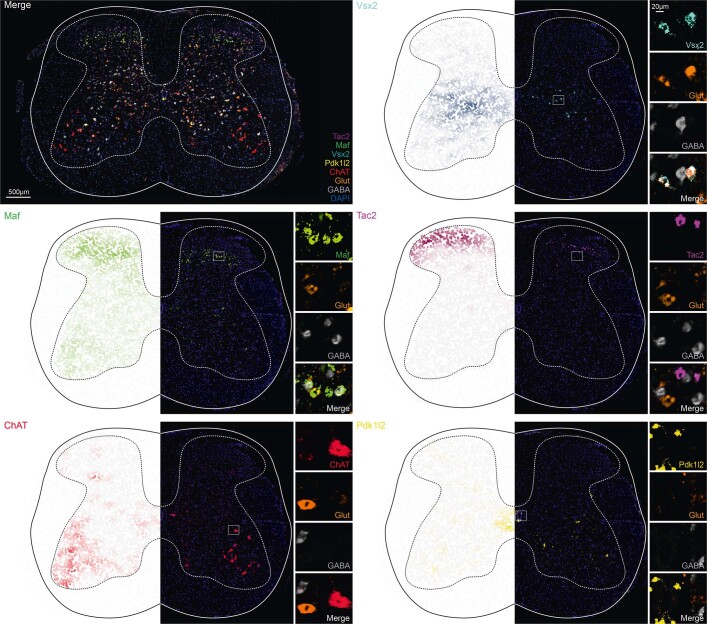
RNAscope ISH confirms the spatial location and neurotransmitter phenotype of five neuron subtypes. Spatial atlases of 5 neuronal subpopulations (Vsx2, Maf, Tac2, Chat and Pkd1l2) after deconvolution of spatial barcodes using snRNA-seq data and corresponding RNAScope ISH labelling for neuron subtype marker and neurotransmitter phenotypes Slc17a6 (Glut) and Slc32a1 (GABA).

We first verified the expected localization of marker genes for dorsal and ventral regions, as well as for canonical neuron subpopulations (Fig. 2c and Extended Data Fig. 6a). We then aimed to localize all the 36 neuron subpopulations from our snRNA-seq data within the lumbar spinal cord by deconvolving the cellular identities of each spatial barcode. We found that neuronal subpopulations identified by snRNA-seq occupied distinct regions of the spinal cord that agreed with their assigned transcriptional identities (Fig. 2c, Extended Data Fig. 6b,c and Supplementary Video 3). We validated the spatial location of key neuronal subpopulations using multiplexed RNAscope (Fig. 2c and Extended Data Fig. 7).

This spatially resolved single-cell atlas of the lumbar spinal cord established a molecular cartography to navigate the uncharted populations of neurons that restore walking after paralysis and thus identify recovery-organizing neurons.

## Identifying recovery-organizing neurons

We next leveraged this comprehensive atlas of the lumbar spinal cord to identify the neuronal subpopulations that are engaged and remodelled by EES^REHAB^.

**Extended Data Fig. 8:**
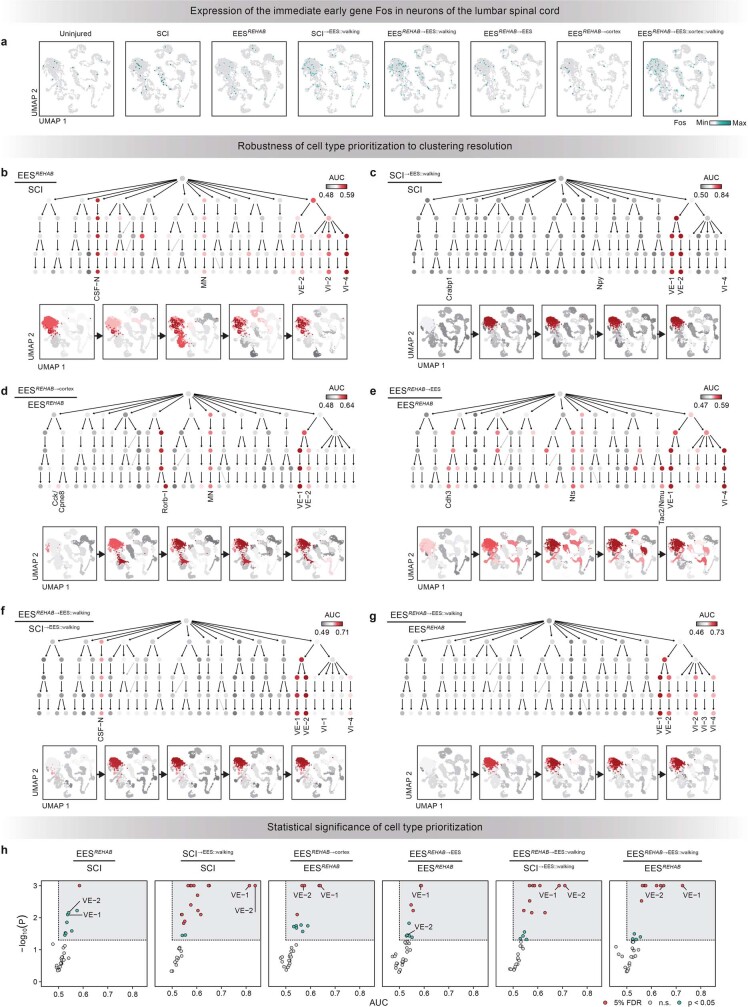
Cell type prioritization to identify recovery-organizing neurons. **a**, Expression of the immediate early gene cFos in neuronal nuclei from each of eight experimental conditions. **b–g**, Robustness of cell type prioritizations across six comparisons capturing the key therapeutic features of EES^REHAB^. Top, cell type prioritizations are shown within a clustering tree of spinal cord neurons declined in five different clustering resolutions, demonstrating the robustness of these prioritizations to the resolution at which transcriptionally defined neuronal subpopulations are defined. The five cell types with the highest AUCs in each comparison are annotated. Bottom, cell type prioritizations over increasingly granular clustering resolutions are visualized on a progression of UMAPs, with neuronal subpopulations colored by the strength of the perturbation response, as inferred by Augur. **h**, Volcano plots showing the strength of the perturbation response (x-axis) and its statistical significance (permutation test, y-axis) for six direct comparisons between two experimental conditions each capturing the key therapeutic features of EES^REHAB^.

We initially sought to identify neuronal subpopulations engaged by EES^REHAB^ on the basis of the upregulation of immediate early genes. However, the low expression of immediate early genes such as cFos in snRNA-seq data impeded this analysis (Extended Data Fig. 8a).

To overcome the shortcoming of single-gene marker analysis, we developed and validated the concept of cell type prioritization^12,13^. We captured this concept in a machine learning method named Augur that identifies cell types that become more transcriptionally separable in response to biological perturbations^12,13^.

**Fig. 3:**
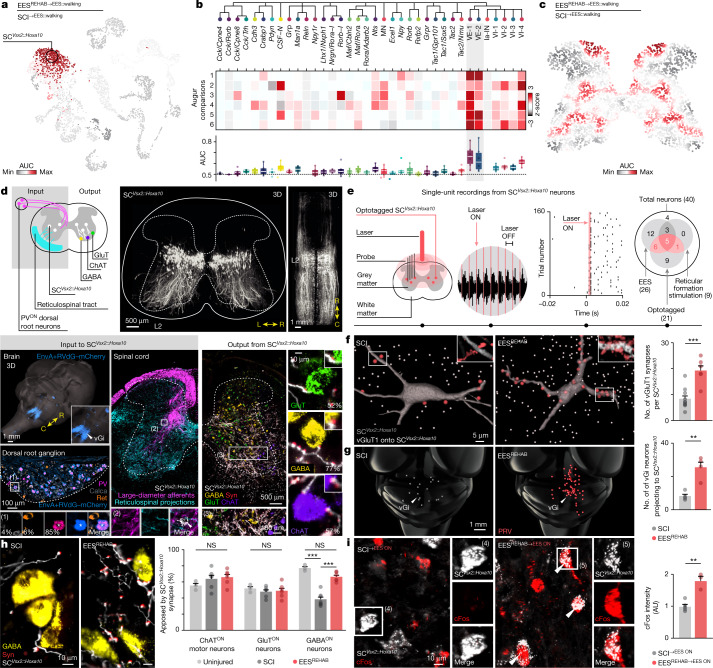
Molecular compass to identify recovery-organizing neurons. **a**, UMAP visualization of 20,990 neurons, coloured by Augur cell type prioritization, identifying perturbation-responsive subpopulations in a representative experimental comparison. **b**, Identification of perturbation-responsive subpopulations across experimental comparisons with Augur. Top, clustering tree of neuronal subpopulations. Middle, heat map showing scaled areas under the curve (AUCs) for individual comparisons: (1) SCI versus SCI^→EES::walking^; (2) SCI versus EES^REHAB^; (3) EES^REHAB^ versus EES^REHAB^^→cortex^; (4) EES^REHAB^ versus EES^REHAB^^→EES^; (5) EES^REHAB^^→EES::walking^ versus SCI^→EES::walking^; (6) EES^REHAB^ versus EES^REHAB^^→EES::walking^. Bottom, distribution of AUCs across all comparisons. **c**, Spatial visualization of 22,127 barcodes in the common coordinate space coloured by Magellan spatial prioritization for the same representative experimental comparison as in **a**. **d**, Synaptic inputs and outputs from SC^*Vsx2::Hoxa10*^ neurons. Inputs: SC^*Vsx2::Hoxa10*^ neurons and their projections. Projections from vGi neurons (AAV5-CAG-COMET-GFP) and large-diameter afferent neurons (PV^cre::Advillin:tdTomato^) onto SC^*Vsx2::Hoxa10*^ neurons. Outputs: synaptic appositions from SC^*Vsx2::Hoxa10*^ neurons (AAV-Dj-hSyn-flex-mGFP-2A-synaptophysin-mRuby) onto glutamatergic, GABAergic and motor neurons. C, caudal; R, rostral; PV, parvalbumin; syn, synaptophysin. **e**, Single-unit recordings of optotagged SC^*Vsx2::Hoxa10*^ neurons. Responses of an optotagged single unit to optogenetic stimulations. Right, the number of units responding to each type of stimulation. **f**, Number of vGluT1 synapses from large-diameter afferents apposing SC^*Vsx2::Hoxa10*^ neurons (*n* = 6 out of 10 mice per group; independent samples two-tailed *t*-test: *t* = 4.9, *P* = 0.002). **g**, Number of reticulospinal neurons projecting to SC^*Vsx2::Hoxa10*^ neurons (*n* = 4 mice per group; independent samples two-tailed *t*-test: *t* = 4.8, *P* = 0.0029). **h**, Percentage of motor neurons, glutamatergic and GABAergic neurons receiving at least one synapse from SC^*Vsx2::Hoxa10*^ neurons (*n* = 6; one-way analysis of variance (ANOVA); choline acetyltransferase (ChAT): *F* = 0.45, *P* = 0.39; glutamatergic: *F* = 0.52, *P* = 0.76; GABAergic: statistics indicate Tukey’s honest significant difference tests: uninjured versus SCI: *P* = 5.2 × 10^−6;^ SCI versus EES^REHAB^: *P* = 1.8 × 10^−5^). **i**, Fluorescent intensity of cFos in *Vsx2*^*cre*^ neurons following walking with EES^ON^ (*n* = 5 out of 3 mice per group; independent samples two-tailed *t*-test: *t* = 5.7, *P* = 0.0013). **f**–**i**, Bars show mean ± s.e.m. with individual points overlaid. AU, arbitrary units. Source data

We hypothesized that the neuronal subpopulations selected during the recovery of walking would be perturbed by each key therapeutic feature of EES^REHAB^. To test this hypothesis, we applied Augur as a molecular compass to prioritize perturbation-responsive neurons within our atlas of the lumbar spinal cord. Strikingly, we found that two populations of excitatory lumbar spinal cord interneurons that expressed *Vsx2* and a marker of caudal spinal cord neurons (*Hoxa10*) (SC^*Vsx2::Hoxa10*^) were prioritized in response to every therapeutic feature of EES^REHAB^ (Fig. 3a,b and Extended Data Fig. 8b–g). This unexpected finding was robust to the resolution used to define neuron subpopulations (Extended Data Fig. 8b–g). The prioritized neurons resembled developmentally defined V2a neurons located in the lumbar spinal cord^35^.

**Extended Data Fig. 9:**
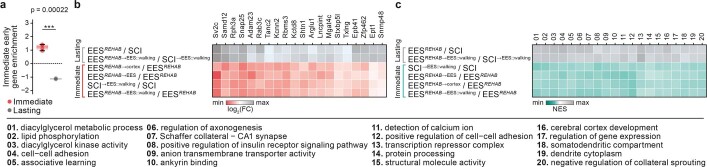
Differential expression analysis of SC^*Vsx2::Hoxa10*^ neurons. **a**, Enrichment of immediate early genes among acutely perturbed SC^*Vsx2::Hoxa10*^ neurons exposed to immediate perturbations (e.g., in response to walking with EES), as compared to chronically perturbed SC^*Vsx2::Hoxa10*^ neurons (e.g., following EES^REHAB^). **b**, Top-ranked genes upregulated in chronically perturbed SC^*Vsx2::Hoxa10*^ neurons, as compared to immediately perturbed neurons. **c**, Top-ranked GO pathways enriched in chronically perturbed SC^*Vsx2::Hoxa10*^ neurons, as compared to immediately perturbed neurons.

We asked whether this prioritization reflected transcriptional changes that were compatible with the immediate recovery of walking with EES and the long-term improvement following EES^REHAB^. We identified an enrichment of immediate early genes within SC^*Vsx2::Hoxa10*^ neurons in response to walking with EES. This transcriptional activity contrasted with the modulation of genes associated with long-lasting structural remodelling following EES^REHAB^ (Extended Data Fig. 9a–c).

**Extended Data Fig. 10:**
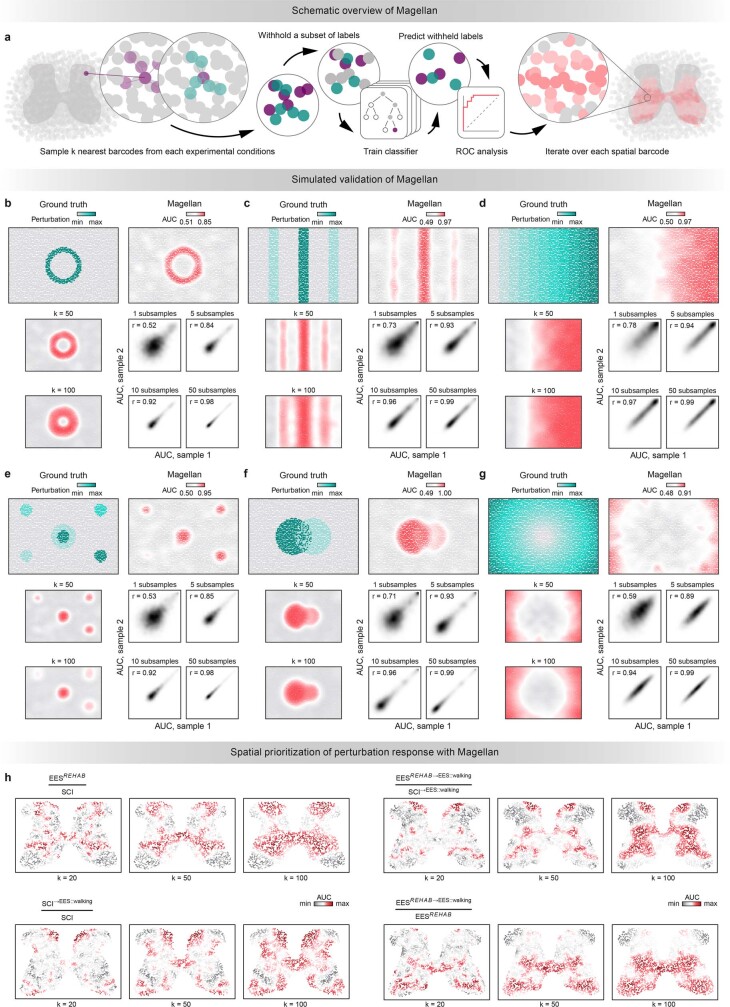
Spatial prioritization of perturbation responses with Magellan. **a**, Schematic overview of Magellan. **b–g**, Validation of Magellan in simulated data. Six different patterns of perturbation were simulated. Perturbation patterns were selected in order to illustrate key desiderata for a robust spatial prioritization method, including the ability to detect sharp borders of perturbation-responsiveness, smooth gradients of perturbation-responsiveness, and multifaceted perturbation responses of differing intensities. Top left, the ground-truth pattern of perturbation responsiveness in simulated spatial transcriptomics data. Top right, spatial prioritizations assigned by Magellan, with default parameters. Bottom left, robustness of spatial prioritizations assigned by Magellan when varying the number of spatial nearest-neighbors, *k*, used to assign a perturbation score to each barcode (default, *k* = 20). The parameter *k* controls the spatial resolution at which Magellan circumscribes the perturbation response. Bottom right, reproducibility of perturbation scores when varying the number of times three-fold cross-validation is repeated for each barcode (default, 50 times). **h**, Robustness of spatial prioritization to the number of spatial nearest-neighbors, *k*, used to assign a perturbation score to each barcode, shown for four direct comparisons between two experimental conditions each capturing key therapeutic features of EES^REHAB^.

We next sought to resolve the spatial topography of this perturbation response. We developed a new method that extends the concept of cell type prioritization to spatial transcriptomics data, which we named Magellan. Magellan uses a spatial nearest-neighbours framework to prioritize perturbation-responsive regions in a biological tissue (Extended Data Fig. 10a). To validate this approach, we generated simulated datasets with spatially complex perturbations. In every scenario, Magellan correctly identified the perturbation-responsive regions (Extended Data Fig. 10b–g).

Within the spinal cord, Magellan circumscribed the walking-associated perturbation response to intermediate laminas, which coincided with the location of SC^*Vsx2::Hoxa10*^ neurons, and to ventral laminas in which neurons producing motor patterns reside (Fig. 3c and Extended Data Fig. 10h).

**Extended Data Fig. 11:**
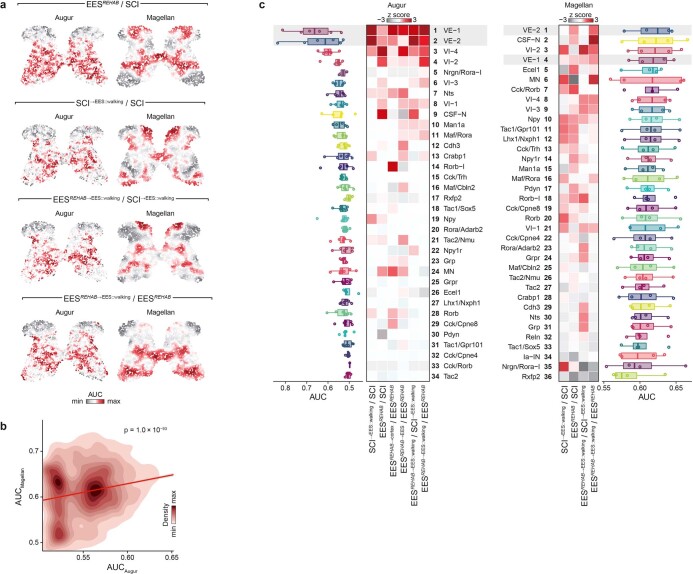
Spatial embedding of single-nucleus transcriptomes. **a**, Comparison of Magellan spatial prioritizations, left, with cell type prioritization scores assigned by Augur within the snRNA-seq dataset and embedded within the common coordinate system of the lumbar spinal cord by Tangram, right. **b**, Correlation between Magellan spatial prioritizations and Augur cell type prioritization scores assigned by Augur within the snRNA-seq dataset and assigned to matching spatial coordinates by Tangram. **c**, Comparison of Augur cell type prioritizations to Magellan spatial prioritizations for barcodes aligned with single-nucleus transcriptomes of each neuronal subtype. Left, Augur cell type prioritizations, as shown in Fig. 3b. Right, mean AUCs assigned by Magellan to spatial barcodes aligned with single-nucleus transcriptomes of each neuronal subtype in the snRNA-seq dataset. Source data

To corroborate the spatial prioritizations assigned by Magellan, we embedded our single-nucleus transcriptomes within the common coordinate system of the mouse spinal cord^36^. We observed a significant correlation between cell type and spatial prioritization scores assigned by Augur and Magellan at matching spatial coordinates (Extended Data Fig. 11a,b). Moreover, we found that the spatial coordinates aligned to SC^*Vsx2::Hoxa10*^ neurons coincided with the most perturbation-responsive regions (Extended Data Fig. 11c).

## Features of SC^*Vsx2::Hoxa10*^ neurons

Cell type and spatial prioritization data implied that excitatory interneurons located in intermediate laminae are the putative neurons that restore walking after paralysis. Therefore, we anticipated that these neurons must possess anatomical and functional features compatible with the key therapeutic features of EES^REHAB^.

**Extended Data Fig. 12:**
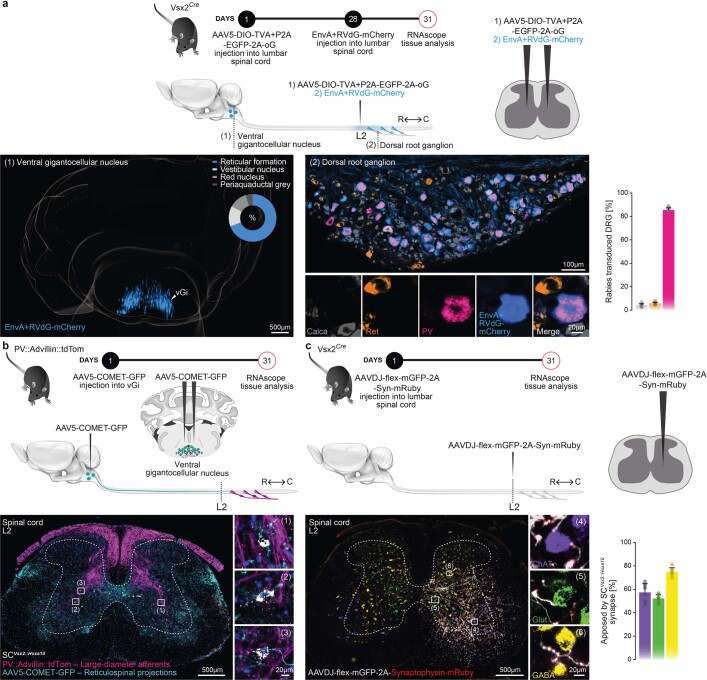
The connectome and projectome of SC^*Vsx2::Hoxa10*^ neurons. **a**, Schematic detailing the experimental set-up to study synaptic inputs to the SC^*Vsx2::Hoxa10*^ neurons. Monosynaptically restricted EnvA pseudotyped and G-protein deleted rabies expressing mCherry revealed reticulospinal neurons in the vGi and PV^ON^ neurons in the DRG give direct inputs to SC^*Vsx2::Hoxa10*^ neurons. **b**, Schematic detailing the experimental set-up to study synaptic inputs to the SC^*Vsx2::Hoxa10*^ neurons. PV^ON^ afferent fibres are labeled in the transgenic PV^Cre::Advillin:tdTomato^ mice. Injections targeted to the vGi labeled descending reticulospinal projections. Vsx2^ON^ neurons were labeled with RNAScope ISH. **c**, Schematic detailing the experimental set-up to study outputs from SC^*Vsx2::Hoxa10*^ neurons. SC^*Vsx2::Hoxa10*^ neurons were traced with AAVDj-hSyn-Flex-mGFPfp-2A-Synaptophysin-mRuby that labels the axons and pre-synaptic terminals. Synaptic appositions from SC^*Vsx2::Hoxa10*^ neurons on to glutamatergic, GABAergic and motor neurons, labeled using RNAScope ISH were quantified. Bar graphs represent the mean with individual data points overlaid. Error bars reflect the standard error of the mean. Source data

**Extended Data Fig. 13:**
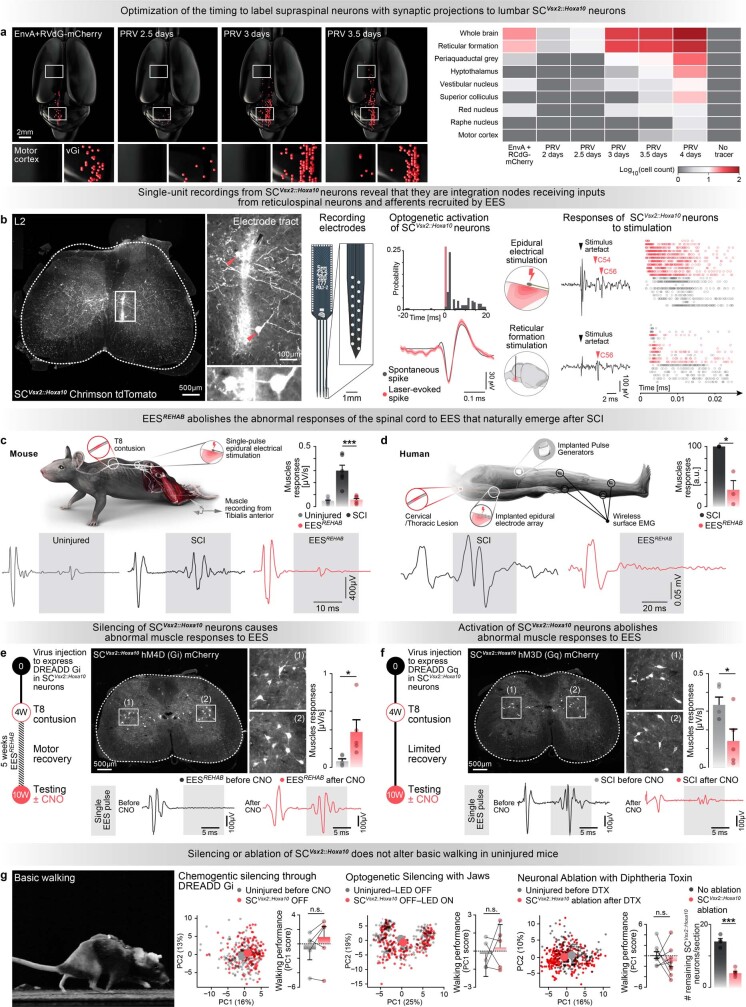
Anatomical and functional features of SC^*Vsx2::Hoxa10*^ neurons. **a**, Optimization of the timing to identify supraspinal neurons connected to SC^*Vsx2::Hoxa10*^ neurons using rabies viruses. Tracing was conducted using infusions of Cre-dependent G-deleted EnvA rabies or Cre-dependent pseudorabies (PRV) into the lumbar spinal cord of Vsx2^Cre^ mice. For pseudorabies experiments, tissue was harvested at 2, 2.5, 3, 3.5 and 4 days after the infusion. Sagittal brain sections were used for 3D reconstructions of brains in Neurolucida, shown here for key timepoints. Quantifications report the number of neurons identified in each region of the brain and brainstem after rabies infusion, and at different timepoints after pseudorabies infusions. At 3.5 days days after pseudorabies injections, neurons were labeled in new brain regions compared to regions labeled with monosynaptically-restricted rabies, suggesting that secondorder neurons became transfected by the pseudorabies. We thus used a window of 3 days to study the connectome of SC^*Vsx2::Hoxa10*^ neurons after SCI. **b**, Transverse spinal cord section from a Vsx2^Cre^ mouse showing Cre-dependent expression of ChrimsonR in SC^*Vsx2::Hoxa10*^ and the tract resulting from the insertion of one electrode shank. Inset shows the cell bodies of SC^*Vsx2::Hoxa10*^ neurons in the vicinity of the tract. The schematic displays the 4-shank, 64-channel silicon probe used for single-unit recordings. The histogram reports the timing of spikes with respect to the onset of photostimulation for the SC^*Vsx2::Hoxa10*^ optotagged single unit shown in Fig. 3d. The waveforms display spontaneous (gray) vs. optogenetically-evoked (red) spikes from an SC^*Vsx2::Hoxa10*^ optotagged single unit. Data are shown as mean traces with standard error of the mean ribbons. The traces on the right were obtained from the same recording site during EES (top) and stimulation of the reticular formation (bottom). In these trials, two single-units responded to EES, whereas only one of these two units also responded to stimulation of the reticular formation (C56). The plot on the right reports the latencies of spikes from SC^*Vsx2::Hoxa10*^ optotagged single units following EES across all trials (150 trials, top) and stimulation of the reticular stimulation (100 trials, bottom). Neurons that consistently responded to stimulations with short latencies (putatively monosynaptic) are highlighted in red. **c**, To evaluate the response of the spinal cord to EES, we measured muscle responses in the tibialis anterior when delivering a single pulse of EES. We tested uninjured mice, mice with chronic SCI, and mice that had undergone EES^REHAB^. Mice with chronic SCI exhibited abnormal long-latency responses (range: 10 to 20 ms), which are highlighted within the grey area and quantified in the bar plots as integral of root mean square (RMS) (*n* = 6 mice per group; statistics indicate Tukey HSD tests of the key comparison following one-way ANOVA, *P* = 0.0001). **d**, The evaluations reported in c for mice were also conducted in the cohort of human participants. Three of the participants (DM002, GO004, HT008) showed abnormal long-latency responses (range: 50 to 100 ms) to single-pulse of EES before EES^REHAB^. EES^REHAB^ nearly completely abolished these responses. The bar plot reports the normalised amplitude of these responses before and after EES^REHAB^ (*n* = 3, trials > 5/session; mixed-effects model, t = –6.40, *P* = 2.9 × 10^–8^). **e, f**, The role of SC^*Vsx2::Hoxa10*^ neurons in the reorganization of muscle responses to EES was evaluated using Cre-dependent expression of Gi, e, or Gq DREADDs in SC^*Vsx2::Hoxa10*^ neurons, f. The timelines summarize the timing of the various experimental procedures. The photographs illustrate the expression of DREADD receptors in SC^*Vsx2::Hoxa10*^ neurons. Long-latency muscle responses to single-pulse of EES were quantified before and 30 min after CNO injections that either silenced (Gi) or activated (Gq) SC^*Vsx2::Hoxa10*^ neurons. Bar plots report the integral of the RMS of long-latency muscle responses for each each experimental condition (*n* = 5 mice per group (Gi); paired samples two-tailed t-test, t = 2.4, *P* = 0.046; *n* = 5 mice per group (Gq); paired samples two-tailed t-test, t = 2.8, *P* = 0.047). **g**, Three complementary strategies were used to evaluate the role of SC^*Vsx2::Hoxa10*^ neurons during basic unskilled walking in uninjured mice: targeted injections of AAV5-flex-hm4di (Gi) or AAV5-flex-Jaws in the lumbar spinal cord of Vsx2^Cre^ mice to evaluate the short-term or immediate impact of silencing SC^*Vsx2::Hoxa10*^ neurons, and targeted injection of AAV-flex-DTR to evaluate the long-term impact of the complete ablation of SC^*Vsx2::Hoxa10*^ neurons. Locomotor performance was evaluated during overground walking using the procedures detailed in Extended Data Fig. 2e (n > 20 gait cycles per mouse, *n* = 5 mice per group). The bar plot reports locomotor performance, quantified as average scores on PC1 (*n* = 5 mice per group except for DTR with *n* = 8 mice per group; paired samples two-tailed t-test, Gi: t = 1.4; *P* = 0.23; LED: t = 1.5, *P* = 0.21; DTR: t = 1.1, *P* = 0.33), and the number of SC^*Vsx2::Hoxa10*^ neurons per tissue section (SC^*Vsx2::Hoxa10*^ ablation, *n* = 7 mice; no ablation, *n* = 4 mice; independent samples two-tailed t-test, t = 12.3, *P* = 6.2 × 10^–7^). Bar plots show the mean with individual data points overlaid. Error bars show the standard error of the mean. *, *P* < 0.05; **, P < 0.01; ***, P < 0.001. Source data

We first asked whether SC^*Vsx2::Hoxa10*^ neurons are endowed with the appropriate anatomical connectome (Extended Data Fig. 12). EES is known to enable walking through the recruitment of large-diameter afferents^37,38^. We also know that reticulospinal neurons from the vGi mediate the volitional modulation of walking during EES^28^. Therefore, we hypothesized that projections from large-diameter afferents and reticulospinal neurons converge onto SC^*Vsx2::Hoxa10*^ neurons (Fig. 3d,e and Extended Data Fig. 13a).

Monosynaptically restricted trans-synaptic tracing revealed that SC^*Vsx2::Hoxa10*^ neurons receive direct synaptic projections from large-diameter afferents of parvalbumin-expressing neurons located in dorsal root ganglia (PV^ON^) and from neurons located in the vGi (Fig. 3d and Extended Data Fig. 12a). Single-unit recordings of optogenetically identified SC^*Vsx2::Hoxa10*^ neurons confirmed that both reticulospinal neuron and large-diameter afferents elicit short-latency responses in a subset of the same SC^*Vsx2::Hoxa10*^ neurons (Fig. 3e and Extended Data Fig. 13b). Consistent with this functional connectivity, we found that EES^REHAB^ increased the density of synaptic projections from large-diameter afferents and reticulospinal fibres onto SC^*Vsx2::Hoxa10*^ neurons (Fig. 3f,g).

We speculated that SC^*Vsx2::Hoxa10*^ neurons would also project to neurons involved in the production of walking. We found that SC^*Vsx2::Hoxa10*^ neurons exclusively project to the ventral spinal cord, where they establish dense synaptic appositions onto 52% of glutamatergic (GluT^ON^), 77% of GABAergic (GABA^ON^) and 56% of cholinergic (ChAT^ON^) neurons located in the ventral spinal cord (Fig. 3d and Extended Data Fig. 12c). SCI induced a pronounced reduction in the density of synaptic appositions from SC^*Vsx2::Hoxa10*^ neurons onto ventrally located GABA^ON^ neurons. This reorganization was not observed in mice that had undergone EES^REHAB^ (Fig. 3h).

We next reasoned that if EES^REHAB^ mediates activity-dependent selection of SC^*Vsx2::Hoxa10*^ neurons, these neurons must remain activated during walking after EES^REHAB^. Despite the substantial reduction in the number of cFos^ON^ neurons during walking in mice that had undergone EES^REHAB^ (Fig. 1i), we found that this transcriptional activity doubled in SC^*Vsx2::Hoxa10*^ neurons after EES^REHAB^ (Fig. 3i).

We finally surmised that if SC^*Vsx2::Hoxa10*^ neurons are essential for walking after EES^REHAB^, they must modify how the spinal cord responds to EES. To test this hypothesis, we quantified the responses in leg muscles following the application of EES pulses (Extended Data Fig. 13c). We found that SCI led to the development of aberrant long-latency responses that have been linked to poor walking performance in rodents and humans^39^. EES^REHAB^ abolished these responses in both mice and humans (Extended Data Fig. 13c,d). To assess whether SC^*Vsx2::Hoxa10*^ neurons contribute to these responses, we manipulated their activity using chemogenetics. Inactivation of SC^*Vsx2::Hoxa10*^ neurons after EES^REHAB^ phenocopied the emergence of aberrant responses observed in mice with chronic SCI (Extended Data Fig. 13e), whereas their activation in mice with SCI abolished these responses (Extended Data Fig. 13f).

We concluded that SC^*Vsx2::Hoxa10*^ neurons located in the intermediate laminae of the lumbar spinal cord possess the anatomical and functional properties that are compatible with the key therapeutic features of EES^REHAB^.

## The neurons that restore walking

We next aimed to determine whether SC^*Vsx2::Hoxa10*^ neurons are necessary and sufficient to restore walking after paralysis.

**Extended Data Fig. 14:**
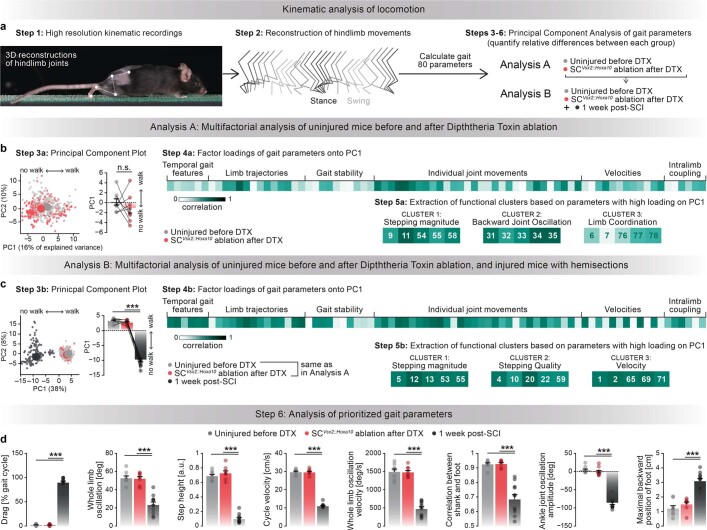
Gait analysis of uninjured mice after diphtheria toxin ablation of SC^*Vsx2::Hoxa10*^ neurons. **a**, Workflow of kinematic gait analysis. **Step 1:** Locomotor performances of uninjured mice were evaluated during quadrupedal walking on a treadmill. Bilateral leg kinematics were captured with twelve infrared cameras that tracked reflective markers attached to the crest, hip, knee, ankle joints and distal toes. **Step 2:** The limbs were modelled as an interconnected chain of segments and a total of 78 gait parameters were calculated from the recordings. All gait parameters are reported in Supplementary Table 2. **Step 3–6:** To evaluate differences between experimental conditions, as well as to identify the most relevant parameters to account for these differences, we implemented a multistep multifactorial analysis based on principal component analysis, which we described in detail previously^29,72^. **b**, Multifactorial analysis including the experimental groups of uninjured mice before and after diphtheria toxin ablation. **Step 3a**: In the principal component analysis plot, each dot represents a gait cycle (*n* = 20 per mouse, *n* = 8 mice per group). Larger dots represent the mean of each experimental group. The first principal component (PC1) usually distinguishes the largest differences in gait. In this case, only minimal non-significant differences were detected. The barplots quantify locomotor performances as the scores on PC1 (*n* = 8; paired samples two-tailed t-test; t = 0.8874; *P* = 0.4043). **Step 4a:** Analysis of factor loadings on PC1 reveal potential differences in 3 functional gait clusters (**Step 5a**). **c**, As in **b**, but including an additional group of mice that underwent hemisection injuries. Only the gait data from ipsilesional hindlimb are included in the analysis. Please note the obvious differences on PC1 and the higher correlation of gait parameters onto PC1 (*n* = 8; statistics indicate Tukey HSD tests following repeated measures one-way analysis of variance (ANOVA); *P* = 1.7 x 10^−8^). **d**, The barplots quantify key gait parameters found in the gait analysis in **b-c**. Bars report n = 8 mice per group, statistics indicate Tukey HSD tests following repeated measures ANOVA, *P* = 8.9 x 10^−11^, *P* = 2.7 x 10^−6^, *P* = 1.2 x 10^−8^, *P* = 2.5 x 10^−13^, *P* = 2.4 x 10^−8^, *P* = 6.4 x 10^−4^, *P* = 9.8 x 10^−8^ and *P* = 6.9 x 10^−7^, respectively. Bar graphs represent the mean with individual data points overlaid. Error bars reflect the standard error of the mean.  *, **, *** indicate *P* < 0.05, 0.01, and 0.001, respectively. Source data

We first tested whether SC^*Vsx2::Hoxa10*^ neurons are necessary for the production of walking in healthy mice. Optogenetic and chemogenetic inactivation, as well as targeted ablation of SC^*Vsx2::Hoxa10*^ neurons, had no detectable impact on basic walking (Extended Data Figs. 13g and 14 and Supplementary Video 4).

**Fig. 4:**
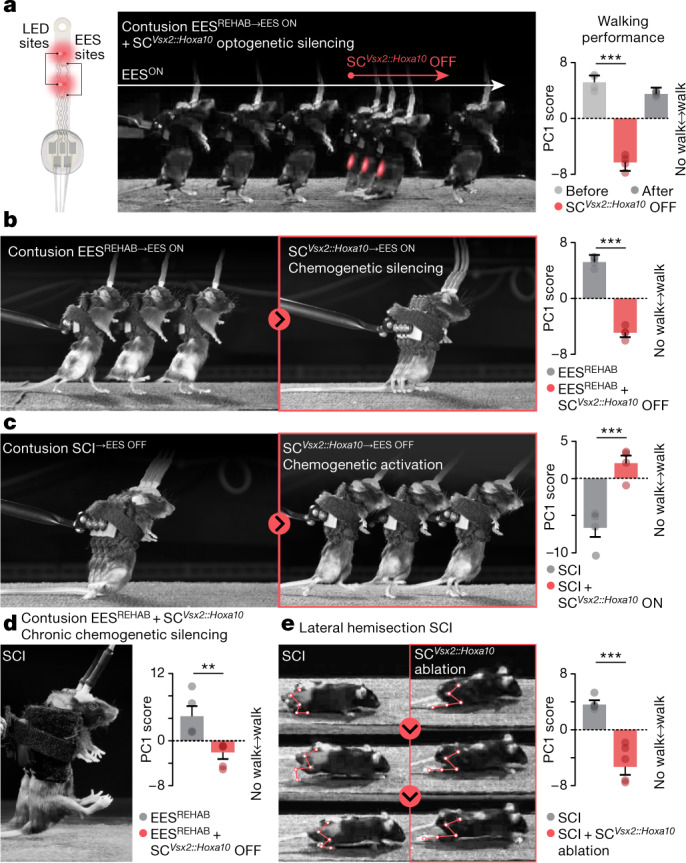
SC^*Vsx2::Hoxa10*^ neurons are required for the recovery of walking after paralysis. **a**, Implantable optoelectronic device to deliver EES and photostimulation. After EES^REHAB^, chronophotography of walking during EES^ON^ in a representative mouse. Red-shifted light is delivered for a few seconds to silence SC^*Vsx2::Hoxa10*^ neurons (AAV5-Syn-flex-ChrimsonR-tdTomato; *n* = 4; Tukey’s honest significant difference tests following repeated measures one-way analysis of variance (ANOVA): *P* = 0.0023). **b**, Chronophotography of walking after EES^REHAB^ in a representative mouse with EES^ON^ before and after chemogenetic silencing of SC^*Vsx2::Hoxa10*^ neurons (AAV5-hSyn-DIO-hm4D-(Gi)-mCherry; *n* = 4; paired samples two-tailed *t*-test; *t* = −21.3, *P* = 0.0002). **c**, Chronophotography of walking in representative mice with no EES^REHAB^, with EES^OFF^ before and after chemogenetic activation of SC^*Vsx2::Hoxa10*^ neurons (AAV5-hSyn-DIO-hm3D-(Gq)-mCherry; *n* = 4; paired samples two-tailed *t*-test; *t* = 5.3, *P* = 0.0013). **d**, After EES^REHAB^, chronophotography of walking in representative mice with EES^ON^. SC^*Vsx2::Hoxa10*^ neurons were silenced during the entire period of EES^REHAB^ (*n* = 5; independent samples two-tailed *t*-test: *t* = −3.5, *P* = 0.008). **e**, Chronophotography of representative mice walking without EES after recovery from a lateral hemisection SCI. Kinematic limb reconstruction is overlaid. Right, same condition, but SC^*Vsx2::Hoxa10*^ neurons located in lumbar segments were ablated before the SCI (AAV5-CAG-flex-DTR; *n* = 5 per group; independent samples two-tailed *t*-test: *t* = 5.9, *P* = 0.0004). **a**–**e**, Bars show mean ± s.e.m. with individual points overlaid. Source data

**Extended Data Fig. 15:**
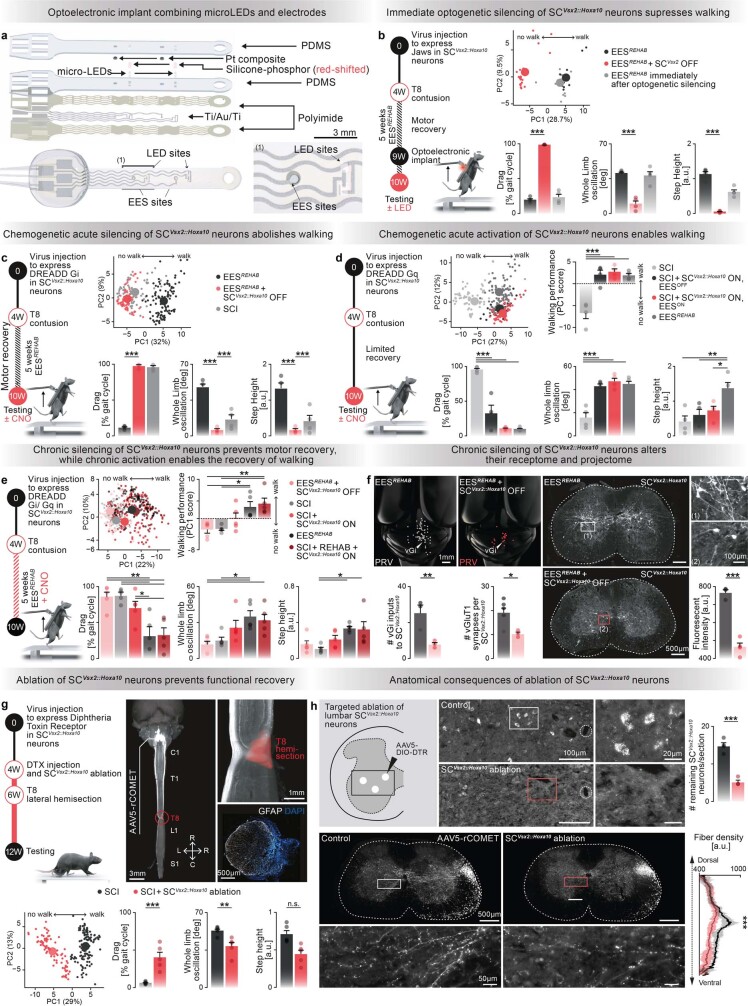
SC^*Vsx2::Hoxa10*^ neurons are necessary and sufficient for the recovery of walking after paralysis. **a**, Exploded diagram of a new wireless optoelectronic system that integrates on the same implant red-shifted microLEDs^40^ to deliver deeply-penetrating photostimulation and electrodes to deliver EES. The zoom shows the microLEDs and electrodes for EES. **b**, Optogenetic silencing of SC^*Vsx2::Hoxa10*^ neurons in mice that underwent four weeks of EES^REHAB^. Experiments and data are shown using the same conventions as in Extended Data Figs. 2 and 13﻿9 (*n* > 20 per mouse, *n* = 4 mice per group). Bars report *n* = 4 mice per group; statistics indicate Tukey HSD tests following repeated measures ANOVA, *P* < 10^–15^, *P* = 0.003 and *P* = 0.002, respectively. **c**, As in **b**, but during acute silencing of SC^*Vsx2::Hoxa10*^ neurons with Gi in mice that underwent four weeks of EES^REHAB^ (*n* = 4 mice per group; statistics indicate Tukey HSD tests following one-way ANOVA, *P* = 7.2 × 10^–10^, *P* = 2.1 × 10^–5^, *P* = 0.0006 respectively). **d**, As in **c**, but during acute activation of SC^*Vsx2::Hoxa10*^ neurons with Gq in mice tested at four weeks post-SCI (no EES^REHAB^) with EES^OFF^ and EES^ON^ (*n* = 4 mice per group; statistics indicate Tukey HSD tests following one-way ANOVA, *P* = 3.1 × 10^–5^, *P* = 4.4 × 10^–7^, *P* = 3.1 × 10^–5^, *P* = 0.0069 respectively). **e**, As in **d**, but following the chronic silencing of SC^*Vsx2::Hoxa10*^ neurons (Gi, CNO in drinking water) in mice that underwent four weeks of EES^REHAB^, chronic activation of SC^*Vsx2::Hoxa10*^ neurons (Gq, CNO in drinking water) in mice after SCI, and chronic activation of SC^*Vsx2::Hoxa10*^ neurons (Gq, CNO in drinking water) in mice that underwent four weeks of rehabilitation without EES (*n* = 5 mice per group; statistics indicate Tukey HSD tests following one-way ANOVA, *P* = 0.0007, *P* = 1.6 × 10^–5^, *P* = 0.004, *P* = 0.011, respectively). **f**, Chronic silencing of SC^*Vsx2::Hoxa10*^ neurons during EES^*REHAB*^ altered the connectome and projectome of SC^*Vsx2::Hoxa10*^ neurons compared to mice that underwent EES^REHAB^. 3D reconstructions show the labeled neurons in the reticular formation 3 days following the infusion of Cre-dependent PRV Ba2017 in the lumbar spinal cord of Vsx2^Cre^ mice. The density of vGluT1 synapses apposing SC^*Vsx2::Hoxa10*^ neurons was quantified to evaluate the synaptic projection from large-diameter afferent fibers onto SC^*Vsx2::Hoxa10*^ neurons. The projectome of SC^*Vsx2::Hoxa10*^ neurons was vizualized using infusions of AAV-flex-tdTomato in the lumbar spinal cord. The bar plots show the average number of vGluT1 synapses apposing SC^*Vsx2::Hoxa10*^ neurons (*n* = 6 mice per group; independent samples two-tailed t-test, t = –3.1, *P* = 0.018), the number of neurons in the reticular formation (*n* = 4 and 3 mice per group, respectively; independent samples two-tailed t-test, t = –4.3, *P* = 0.0076), and the density of projections from SC^*Vsx2::Hoxa10*^ neurons in the ventral horn (*n* = 4 mice per group; independent samples two-tailed t-test, t = –9.2, *P* = 0.0003). **g**, The impact of the chronic ablation of SC^*Vsx2::Hoxa10*^ neurons on the natural recovery of mice with thoracic lateral hemisection SCI was evaluated as in Extended Data Fig. 13g, and summarized in the timeline of experiments. Photographs show coronal sections of the hemisected spinal cord at the lesion epicenter, while CLARITY-optimized light sheet microscopy illustrates the interruption of the reticulospinal tract on the hemisected side. Locomotor performances were evaluated as detailed in the other panels. Bar plots, *n* = 5 mice per group; independent samples two-tailed t-test, t = 6.6, *P* = 0.0002; t = –3.4, *P* = 0.09; t = –1.9, *P* = 0.093, respectively). **h**, Photographs show coronal sections of the spinal cord with Vsx2 RNAScope, confirming the reduction in the number of SC^*Vsx2::Hoxa10*^ neurons in the lumbar spinal cord of Vsx2^Cre^ mice after diphtheria toxin injections. Bar plots report the mean number of Vsx2 labeled neurons per section in the spinal grey matter (SCI *n* = 3, SCI with SC^*Vsx2::Hoxa10*^ ablation *n* = 4 mice; independent samples two-tailed t-test, t = 9.5, *P* = 0.0001). Photographs below show examples of projections from reticulospinal neurons in the lumbar spinal cord in both groups of mice. The plot reports the mean density of reticulospinal projections across the dorsoventral extent of the spinal cord for mice with SCI (*n* = 3) and mice with SCI and ablation of SC^*Vsx2::Hoxa10*^ neurons (*n* = 4). Ribbons show the standard deviation (two-tailed Wilcoxon rank-sum test, W = 2008248, *P* < 10^–15^). Bar plots show the mean with individual data points overlaid. Error bars show the standard error of the mean. *, *P* < 0.05; **, *P* < 0.01; ***, *P* < 0.001. Source data

To interrogate the role of SC^*Vsx2::Hoxa10*^ neurons after EES^REHAB^, we augmented our wireless system for spinal cord optogenetics with electrodes^40^. On the same epidural implant, we integrated both red-shifted microLEDs to deliver deeply penetrating photostimulation and electrodes to enable walking with EES (Extended Data Fig. 15a). Optogenetic inactivation of SC^*Vsx2::Hoxa10*^ neurons in mice with SCI instantly suppressed walking enabled by EES. Walking resumed immediately when the microLEDs were turned off (Fig. 4a, Extended Data Fig. 15a,b and Supplementary Video 5). We confirmed these results using chemogenetic inactivation (Fig. 4b and Extended Data Fig. 15c). Conversely, activation of SC^*Vsx2::Hoxa10*^ neurons with chronic paralysis instantly phenocopied the essential elements of the recovery of walking observed in mice that had undergone EES^REHAB^, regardless of whether EES was switched on or off (Fig. 4c, Extended Data Fig. 15d and Supplementary Video 5). In turn, chronic chemogenetic activation of SC^*Vsx2::Hoxa10*^ neurons in mice that underwent rehabilitation without EES led to a recovery of walking similar to that in mice that underwent EES^REHAB^ (Extended Data Fig. 15e).

Because silencing of SC^*Vsx2::Hoxa10*^ neurons suppressed walking after SCI, we surmised that chronic silencing of these neurons would prevent recovery in response to EES^REHAB^. We validated this prediction with the sustained chemogenetic silencing of SC^*Vsx2::Hoxa10*^ neurons (Fig. 4d, Extended Data Fig. 15e and Supplementary Video 5). This prevention of recovery coincided with an expected retraction of projections from SC^*Vsx2::Hoxa10*^ neurons, combined with the suppression of the increase in the density of reticulospinal and large-diameter afferent projections onto SC^*Vsx2::Hoxa10*^ neurons (Extended Data Fig. 15f).

We finally asked whether the role of SC^*Vsx2::Hoxa10*^ neurons was confined to EES^REHAB^, or if their participation is a fundamental requirement for recovery from SCI. To answer this question, we studied whether the ablation of SC^*Vsx2::Hoxa10*^ neurons impaired the recovery of walking that naturally occurs following a lateral hemisection of the spinal cord. We found that in the absence of SC^*Vsx2::Hoxa10*^ neurons in the lumbar spinal cord, mice failed to fully recover walking after a lateral hemisection SCI (Fig. 4e, Extended Data Fig. 15g and Supplementary Video 5). This failure was paralleled by a reduced growth of residual reticulospinal projections into grey matter territories below the injury (Extended Data Fig. 15h).

These experiments confirmed that the participation of SC^*Vsx2::Hoxa10*^ neurons is a fundamental requirement for the recovery of walking after paralysis.

## Discussion

Here, we show that EES^REHAB^ restored walking and improved the neurological status of nine people with chronic SCI. This recovery demonstrates the therapeutic efficacy of EES^REHAB^ in a large number of people who exhibited neurological deficits spanning the entire range of severities after SCI, thus opening a path for the clinical deployment of this therapy.

This recovery involved an unexpected reduction of the neuronal activity in the lumbar spinal cord during walking. To understand the underlying mechanisms, we developed a preclinical model that recapitulates the key therapeutic features of EES^REHAB^ in humans, including this reduction of neuronal activity. This model enabled us to establish a spatially resolved single-cell atlas of the lumbar spinal cord during recovery from paralysis. Interrogation of this atlas revealed that EES^REHAB^ leads to activity-dependent selection of a single neuronal subpopulation expressing *Vsx2* and the caudal spinal cord specifying transcription factor *Hoxa10*. These neurons originate from a subset of developmentally defined V2a neurons^35,41–45^, which describe a family of neuronal subpopulations distributed along the neuraxis^35,41–45^. Specific subpopulations of V2a neurons in the brainstem, cervical spinal cord and lumbar spinal cord have been implicated in different aspects of motor control^35,41–45^. Here, we demonstrate that SC^*Vsx2::Hoxa10*^ neurons are uniquely positioned to transform information from brainstem locomotor regions and large-diameter afferents into executive commands that are broadcast to the ventrally located neurons responsible for the production of walking. Whereas these neurons are not critical for walking in the absence of SCI, they become recovery-organizing cells after SCI.

These results demonstrate the essential role of SC^*Vsx2::Hoxa10*^ neurons in orchestrating recovery from paralysis. However, numerous neuronal subpopulations in the brain and spinal cord must also contribute to the production and recovery of walking^16,44,46–48^. For example, ventral inhibitory neurons (V1 and V2b neurons^49,50^), which are located downstream to SC^*Vsx2::Hoxa10*^ neurons, were prioritized in specific comparisons involving the long-term recovery of walking following EES^REHAB^. These neurons are likely to coincide with GABA^ON^ neurons that receive a twofold increase in the density of projections from SC^*Vsx2::Hoxa10*^ neurons following EES^REHAB^. This reorganization may contribute to the overall decrease in the number of transcriptionally active neurons during walking in response to EES^REHAB^.

Understanding the contributions of each neuronal subpopulation to complex behaviours such as walking is a fundamental challenge in neuroscience. Here, we describe unbiased methodologies that leverage comparative single-cell genomics to circumscribe the cellular and spatial origin of perturbation responses. The application of Augur and Magellan to single-nucleus and spatial transcriptomes provides a generalizable framework to prioritize cellular subpopulations in any biological tissue, in response to any biological stimulus.

## Methods

### Study design and participants

All experiments were carried out as part of the ongoing clinical feasibility study STIMO, which investigates the effects of spatiotemporal EES combined with weight-supported overground locomotor training on the recovery of motor function after SCI (clinicaltrials.gov, NCT02936453). The context, primary and secondary endpoints, and timeline of the study are described in Supplementary Note 1. This study was approved by the Swiss ethical authorities (Swissethics protocol number 04/2014 ProjectID: PB_2016-00886, Swissmedic protocol 2016-MD-0002) and was conducted in accordance with the Declaration of Helsinki. All participants provided written informed consent prior to their participation. All surgical and experimental procedures were performed at the Lausanne University Hospital (CHUV) and have been previously described in detail^1^. The study involved assessments before surgery, the surgical implantation of the neurostimulation system, a one-month period during which EES protocols were configured, and a five-month rehabilitation period with physiotherapists taking place four to five times per week for one to three hours. The rehabilitation programme was personalized based on the participants’ improvements. At the end of the rehabilitation period, the participants were given the opportunity to be enroled in a study extension phase during which they could continue using the neurostimulation system at home. They are currently followed-up on a regular basis by the study team for up to six years. To date, a total of nine individuals completed the main part of the study. Their neurological status was evaluated according to the International Standards for Neurological Classification of Spinal Cord Injury (ISNCSCI), and is reported in Supplementary Table 1. The outcomes from these nine individuals are reported in this report.

### EES technologies

In humans, EES was delivered using a paddle lead that was surgically implanted over the epidural surface of the lumbar spinal cord. The first 6 participants were implanted with the Specify 5-6-5 paddle lead, which was originally designed to target the dorsal column in order to alleviate pain (Specify 5-6-5, Medtronic). The last three participants were implanted with a new paddle lead that was specifically developed to target the dorsal roots involved in the control of trunk and leg movements^18^ (ONWARD Medical). The paddle leads were connected to an implantable pulse generator (IPG) (Medtronic Activa RC, Medtronic) commonly used for deep-brain stimulation therapies. We upgraded this IPG with a wireless communication bridge that enabled real-time control over the parameters of EES protocols.

### FDG-PET/CT

Since the participants were equipped with implanted medical devices that were not approved for MRI, we measured the spinal cord glucose consumption as a surrogate of neuronal activity during walking using non-invasive FDG-PET–computed tomography (PET/CT) imaging after walking. This technique was previously used to study the effect of spinal cord stimulation at the cervical level^52^. PET scans were acquired four weeks after the surgical implantation of the devices and after EES^REHAB^ with EES^ON^, using 150 MBq of FDG. Participants were asked to walk for the first 15 min of the FDG uptake. If participants were not able to walk prior to rehabilitation, they were asked to hold onto a Taurus and attempt to walk while their legs were moved by physiotherapists. Spinal cord FDG uptake was then measured at 45 min after radiotracer injection with 3 min per bed position, and assessed in SUV_bw_, from T10 to L2 vertebral body levels using CT images for anatomic localization. The SUV_bw_ index represents the FDG uptake by the neuron cell bodies and dendritic processes forming the grey matter, corrected for injected radiotracer activity, radioactive decay and body weight^53^. Glucose consumption was compared before and after EES^REHAB^ using a mixed effect linear model. PET images were also coregistered in a preoperative 3D MRI scan of the spinal cord for visualization purposes.

### ISNCSCI

Neurological status was assessed based on the ISNCSCI^54^, a comprehensive clinician-administered neurological examination of residual sensory and motor function quantifying SCI severity.

### EES^REHAB^ programme

Participants followed a neurorehabilitation programme four to five times per week for five months. The programme was personalized to participants’ performance, but generally comprised a mixture of walking on a treadmill and overground with multiple assistive devices, sit-to-stand, standing, leg and trunk muscle exercises, swimming and cycling. Activity-specific stimulation programmes were delivered to enable the practice of these activities. The minimum amount of body weight support required to walk overground was recorded during monthly gait assessments.

### Six-minute walk test

Endurance was assessed by the distance covered overground within six minutes with a standard four-wheel walker but without any external assistance^55,56^. This test was performed before and after EES^REHAB^ with EES^ON^ and EES^OFF^.

### Ten-metre walk test

Walking speed was assessed by a timed ten-metre walk test without any external assistance^56^. Only two participants were evaluated with 5% body weight support for safety (ST006, MB007). Participants were instructed to walk with the preferred assistive device as fast as they could. This test was performed before and after EES^REHAB^ with EES^ON^ and EES^OFF^.

### Recordings of muscle activity

Participants were lying relaxed in supine position on an examination table. Electromyographic (EMG) activity was recorded bilaterally from the iliopsoas (Il), rectus femoris (RF), vastus lateralis (VLat), semitendinosus (ST), tibialis anterior (TA), medial gastrocnemius (MG) and soleus (Sol) muscles with wireless bipolar surface electrodes (Myon 320, Myon or Delsys Trigno). Each electrode was placed centrally over the muscle with a longitudinal alignment and an inter-electrode distance of 3 cm. Abrasive paste (Nuprep, 4Weaver and Company) was used for skin preparation to reduce electrode-skin resistance and improve EMG signal quality. Stimulation artefacts required for responses alignment were picked up by an additional pair of surface-EMG electrodes placed over the spine at the thoracolumbar junction or from the iliopsoas sensors ipsilateral to the IPG. Muscular recordings were acquired with Delsys Trigno Plugin v2.0.2 integrated in Nexus v1.8.5. Continuous EMG signals were sampled at 5 kHz (Myon) or 2 kHz (Delsys) and saved to a desktop computer. EMG signals were bandpass filtered between 20 and 450 Hz. Recordings were performed with graded stimulation amplitudes. Each contact of the implanted electrode array was stimulated in monopolar configuration as the cathode, with the case of the implantable pulse generator set as the anode. First, low-amplitude stimulation was applied to identify the lowest response threshold across all recorded muscles (motor threshold (MT)). Then, EES amplitude was increased manually to identify the amplitudes at which the responses reached a plateau, limited to levels that did not cause discomfort to the participant. Finally, single-pulse EES at amplitudes ranging from response threshold to saturation was performed automatically, with four repetitions at each EES amplitude. Recorded EMG responses were segmented in windows from 6–50 ms and from 50–100 ms for short and long-latency components of muscle responses. The amplitudes of each component were quantified as peak-to-peak. Ten long-latency stimulation responses in the range of 1.1–1.3 × MT were quantified.

### Mouse model

Adult male or female C57BL/6 mice (15–35 g body weight, 12–30 weeks of age) or transgenic mice were used for all experiments. *Vglut2*^*cre*^ (Jackson Laboratory 016963), Ai65(RCFL-tdT) (Jackson Laboratory 021875), Parvalbumin (PV)^cre^ (Jackson Laboratory 017320), Advillin^FlpO^ (a gift from V. Abraira) and *Vsx2*^*cre*^ (MMMRRC 36672, also called *Chx10*^*cre*^) transgenic mouse strains were bred and maintained on a mixed genetic background (C57BL/6). Housing, surgery, behavioural experiments and euthanasia were all performed in compliance with the Swiss Veterinary Law guidelines. Mice were maintained in house under standard housing conditions (12-h light/dark cycle) with 24 h access to water and standard chow diet at temperature at 21 ± 1 °C and relative humidity at 55 ± 5%. All procedures and surgeries were approved by the Veterinary Office of the Canton of Geneva. Manual bladder voiding and all other animal care was performed twice daily for the first three weeks after injury and once daily for the remaining period of experiment. All procedures and surgeries were approved by the Veterinary Office of the Canton of Geneva (Switzerland; license GE/25/17).

### Viruses and virus production

Viruses used in this study were either acquired commercially or produced locally. The following AAV plasmids were used and detailed sequence information is available as detailed or upon request: AAVDj-hSyn-flex-mGFP-2A-synaptophysin-mRuby^57^ (Stanford Vector Core Facility, reference AAV DJ GVVC-AAV-100), AAV5-CAG-DIO-COMET-tdTomato and AAV5CAG-COMET-GFP (a gift from M. Tuszynski), AAV5-Syn-flex-ChrimsonR-tdT (Addgene 62723), AAV5-CAG-flex-Jaws-KGC-GFP-ER2 (Addgene 84445), AAV5-hSyn-DIO-hm4D (Gi)-mCherry (Addgene 44362), AAV5-hSyn-DIO-hm3D (Gq)-mCherry (Addgene 44361), AAV5-CAG-flex-tdTomato (a gift from S. Arber), AAV5CAG-flex-human diphtheria toxin receptor (DTR) (a gift from S. Arber), AAV5-DIO-TC66T-2A-eGFP-2A-oG (GT3) (Salk Institute) and AAV5-hSyn-DIO-TVAP2A-EGFP-2A-oG (a gift from T. Karayannis). All floxed AAV vectors used in the present study showed transgene expression only upon Cre-mediated recombination. Trans-synaptic tracings were performed with EnvA-G-deleted rabies–mCherry (GT3) (Salk Institute 32646) or the cre-dependent PRV Ba2017 (expressing GFP; 4.9 × 10^9^ pfu per ml; Princeton University). Injection volumes, coordinates and experimental purpose are described below.

### Surgical procedures

General surgical procedures have been described previously in detail^28^. Surgeries were performed under aseptic conditions and under 1–2% isoflurane in 0.5–1 l min^−1^ flow of oxygen as general anaesthesia. After surgeries, mice were allowed to wake up in an incubator. Analgesia—buprenorphine (Essex Chemie, 0.01–0.05 mg kg^−1^ subcutaneous injection) or rimadyl (5 mg kg^−1^ subcutaneous injection)—was given twice daily for 2–3 days after surgery.

### SCI models

Spinal cord contusion and lateral hemisections were performed as previously described^28^. A laminectomy was made at the mid-thoracic level (T8 and T9 vertebra). To perform a contusion injury, we used a force-controlled spinal cord impactor^58^ (IH-0400 Impactor, Precision Systems and Instrumentation), as previously described^28^. The applied force was set to 95 kDyn. Hemisections were performed at the mid-thoracic level after a laminectomy (T8 vertebra) using a microscalpel.

### Spinal injections

For lumbar cord injections for viral vector tracings, a laminectomy of T13 was performed and 4 injections were made on either side. For lumbar cord injections, which are followed by EES or spinal micro-array implantations, the interlaminar spaces between T12/T13, T13/L1 and L1/L2 were dissected. Injections were performed using a pulled glass pipette driven with the Nanoliter pump (Nanoliter 2010 injector, World Precision Instruments) fixed to a stereotaxic frame. Two injections were made on either side of the spinal cord per interlaminar space. Fifty nanolitres at a rate of 100 nl s^−1^ were injected at 0.6 mm and at 0.3 mm below the dorsal surface of the spinal cord.

### Brain injections

An incision was made across the skull. To target corticospinal neurons in layer V motor cortex, bregma was identified and a craniotomy 1 mm–2 mm medial and −0.5 mm to 2 mm rostro-caudally was performed with a hand-held drill^28^. One-hundred nanolitre injections at 3 nl s^−1^ were made bilaterally at medial–lateral 1.2 mm and 1.7 mm, rostro-caudally at 0 mm, −0.5 mm, −1 mm and −1.5 mm, dorso-ventrally at a depth of 0.5 mm from the brain surface. To target descending neurons in the reticulospinal formation, bregma was identified and a craniotomy 5 mm-6 mm dorsal and 0 mm–2 mm lateral to Bregma was performed^28^. One-hundred nanolitre injections at 3 nl s^−1^ were made bilaterally at medial–lateral 0.3 mm, rostro-caudally at −5.8 mm and −6.2 mm, dorso-ventrally at a depth of 5.6 mm from the brain surface.

### EES implantations and stimulation protocols

All the procedures have been detailed previously^3–5,28,59,60^. To position electrodes to deliver EES in mice, laminotomies (removal of only the connective tissue in between the bones, but not bones) were performed at T12/T13 and L1/L2 to expose the spinal cord. Tefloncoated stainless steel wires connected to a percutaneous connector (Omnetics Connector Corporation) were inserted on either side and passed between the spinal cord and the vertebral bones to the other opening. A small part of insulation was removed and the exposed stimulation sites were positioned over L2 and S1. A common ground was inserted subcutaneously. The percutaneous connector was cemented to the skull. Stimulation was therefore delivered to both sites simultaneously. Conventional stimulation protocols consisted of continuous EES delivered at 40 Hz with 0.2 ms pulses at 50–300 µA. High-frequency burst stimulation protocols consisted of 10-ms-long bursts of 0.2-ms-long pulses, 50–300 µA at 600 Hz with a modulating frequency of 30 Hz. This high-frequency burst stimulation protocol was subsequently used for all acute and chronic experiments.

### Photostimulation of the motor cortex

All the procedures have been detailed previously^25–28^. In the same surgical procedure as brain injections into the motor cortex, optic fibres (200 µm core diameter, 9.22 NA, Thorlabs) passed through 1.25 mm ceramic ferrules (CFLC126-10, Thorlabs) were implanted bilaterally at 0.5 mm lateral and 0.5 mm caudal to Bregma at a depth of 0.5 mm. Three screws (AMS120/5B25, Antrin Miniature Specialties) were inserted into the skull, surrounding the ferrules connector. Fresh dental cement was then poured around the screws and ferrules, and left until cured. Light was transmitted through a ferrule-to ferrule connection. A blue laser (laserglow, 473 nm Blue DPSS Laser System) was used to photostimulate the cortex every 5 s over 3 s and consisted of 10 ms long pulses^28^ at 40 Hz.

### Chronic implantation of electrodes to record muscle activity

All the procedures have been detailed previously^3–5,28,60^. An incision over the muscle of interest (tibialis anterior, gastrocnemius) was made and if needed the muscle was exposed by blunt dissection of overlying tissue. Bipolar intramuscular electrodes were inserted into the muscle parallel to the muscle fibre orientation. To confirm optimal placing, the wires were electrically stimulated resulting in a muscle. Electrodes were fixed in place by suturing either side of the electrode that exited the muscle 45. A grounding wire was implanted subcutaneously. Wires were connected to a percutaneous connector (Omnetics Connector Corporation) cemented to the skull. Electromyographic signals (2 kHz) were amplified (1k), filtered (100–1k bandwidth, A-M Systems Differential AC Amplifier Model 1700) and digitalized either with the Vicon System or with the Powerlab system (AdInstruments).

### EES^REHAB^ in mice

One week after contusion injury, mice were trained daily for four weeks. Five minutes before training, the mice received a small bolus of quipazine (5-HT2A/C, 0.2–0.3 mg kg^−1^) and intraperitoneal injections of 8-OH-DPAT (5HT1A/7, 0.05-0.2 mg kg^−1^) to reactivate the lumbar spinal neurons and to enable sustained locomotion during neurorehabilitation^4^. The dose was progressively reduced during recovery. EES^REHAB^ consisted of a combination of bipedal stepping on a treadmill with adjustable robotic body weight support (9 cm s^−1^, Robomedica) and overground quadrupedal walking supported in a multidirectional robotic body weight support interface^24^. EES was applied throughout the period of neurorehabilitation. Sessions lasted between 30 and 40 min.

### Experimental conditions

Different experimental conditions used throughout the project are summarized in Fig. 2a. Mice were divided in three experimental groups: uninjured, SCI (no neurorehabilitation), and EES^REHAB^. At the end of the experimental period, mice were terminated to harvest fresh tissues or perfused and dissected. Half of the groups of mice underwent a terminal experimental condition immediately before being terminated, which is denoted by ^→final_condition^. If more than one component was integrated in the terminal experimental condition, the additional component is denoted by ^::^. Group 1 (uninjured) consisted of uninjured mice that did not perform a terminal behavioural task. Mice in group 2 (SCI) received a contusion SCI and did not perform a terminal behavioural task. Mice in group 3 (EES^REHAB^) received a contusion SCI and followed EES^REHAB^ for 4 weeks, but did not perform a terminal behavioural task. Mice in group 4 (SCI^→EES::walking^) received a contusion SCI and walked with EES^ON^ for 30 min immediately before being terminated. Mice in group 5 (EES^REHAB^^→EES::walking^) received a contusion SCI and followed EES^REHAB^ for 4 weeks, and walked with EES^ON^ for 30 min immediately before being terminated. Mice in group 6 (EES^REHAB^^→EES^) received a contusion SCI and followed EES^REHAB^ for 4 weeks, and were stimulated for 30 min with EES just below motor threshold immediately before being terminated. Mice in group 7 (EES^REHAB^^→cortex^) received a contusion SCI and followed EES^REHAB^ for 4 weeks, and walked with optogenetic stimulation of the motor cortex for 30 min immediately before being terminated. Mice in group 8 (EES^REHAB^^→cortex::walking^) received a contusion SCI and followed EES^REHAB^ for 4 weeks, and walked with EES^ON^ and optogenetic stimulation of the motor cortex for 30 min immediately before being terminated (see Supplementary Table 3).

### Behavioural assessments

All the behavioural procedures have been described in detail previously^4,40^. Locomotor performances of uninjured mice and mice with lateral hemisection SCI were evaluated during quadrupedal walking on a treadmill. Locomotor performances of mice with contusion SCI were evaluated during walking bipedally on a treadmill or during quadrupedal overground walking. All the final behavioural assessments of mice with contusion SCI were performed with EES^OFF^ and repeated with EES^ON^, but in the absence of 5-HT agonists. Bilateral leg kinematics were captured with twelve infrared cameras of a Vicon Motion Systems that tracked reflective markers attached to the crest, hip, knee, ankle joints and distal toes. The limbs were modelled as an interconnected chain of segments and a total of 80 gait parameters were calculated from the recordings. All gait parameters are reported in Supplementary Table 2. To evaluate differences between experimental conditions, as well as to identify the most relevant parameters to account for these differences, we implemented a multistep multifactorial analysis based on principal component analysis, which we described in detail previously^28,61,62^ (Extended Data Fig. 14). In brief, for each experiment dataset, the principal component analysis was performed by computing the covariance matrix *A* of the ensemble of parameters over the gait cycle, after subtraction of their respective mean values. The principal components were computed from eigenvalues *λ*_j_ and eigenvectors *U*_j_ of *A*. The principal components were ordered according to the amount of data variance accounted for by each component. The coordinate of each gait cycle on the first principal component—that is, the component vector explaining the greatest amount of variance across the gait parameters, was thereafter referred to as the walking performance.

### Chronophotography

Chronophotography was used to generate representative series of still pictures arranged in a single photograph to illustrate the locomotor abilities of mice. Videos at 25 fps or photographs at 15 fps were recorded while mice were performing locomotor tasks such as quadrupedal or bipedal walking on a treadmill or runway. Images from these recordings were chosen to best illustrate the different consecutive phases of walking of the hindlimbs−that is, stance and swing phases. The frequency of chosen pictures varied due to the varying velocity of the mice. The series of pictures were assembled in Photoshop while blending out non-essential detail.

### Neuron-specific ablation and chemogenetics

For ablation experiments with the diphtheria toxin, AAV-flex-DTRV was infused in the lumbar spinal cord of *Vsx2*^*cre*^ mice^63^. Four weeks after spinal infusions, mice received intraperitoneal injections of diphtheria toxin (Sigma, D0564) diluted in saline (100 µg kg^−1^) to ablate SC^*Vsx2::Hoxa10*^ neurons. Mice were tested just before ablation and two weeks post-ablation. To manipulate the activity of SC^*Vsx2::Hoxa10*^ neurons, AAV5-hSyn-DIO-hm4D or AAV5hSyn-DIO-hm3D^64^ were infused in the lumbar spinal cord of *Vsx2*^*cre*^ mice four weeks prior to any behavioural experiments or SCI. On the day of the experiment, mice were tested on the behavioural task immediately before and between 30–45 min after intraperitoneal injections of 5 mg kg^−1^ clozapine *N*-oxide (CNO) (Carbosynth, CAS: 34233-69-7, suspended in 2% DMSO in saline)^28^. For chronic chemogenetic silencing, AAV5-hSynDIO-hm4D or AAV5-hSyn-DIO-hm3D was infused in the lumbar spinal cord of *Vsx2*^*cre*^ mice four weeks prior to the SCI. Mice received approximately 0.05 mg ml^−1^ CNO with 5 mM sucrose^65^ in their drinking water each day, which amounted to approximately 5 mg kg^−1^ CNO per day. Experiments that involved chronic chemogenetic activation or silencing of SC^*Vsx2::Hoxa10*^ neurons were performed two days after the CNO was removed from the drinking water.

### Recording of muscle activity

A subset of mice that had EES electrodes implanted over the lumbar spinal cord were assessed with electrophysiology. Mice were anaesthetized with ketamine/xylazine. Ketamine maintenance doses were administered as needed. Two needle electrodes were inserted in the tibialis anterior muscle. Single-pulse stimulation of 200-µs-long pulses delivered every 5 s was applied to the dorsal surface of the spinal cord and muscle responses were recorded for 50 ms post-stimulation. The pulses (resulting in a stimulus artefact) were aligned to 0 ms, as shown in all figure panels (Extended Data Fig. 13). Stimulation consistently produced short-latency responses (likely to be monosynaptic responses) and long-latency responses (likely to be polysynaptic responses). We calculated the root mean squares followed by the integrals for 2–7 ms (early response) and 10–20 ms (long-latency response). Bar graphs report the long-latency muscle responses.

### Perfusions

Mice were perfused at the end of the experiments. Mice were deeply anaesthetized by an intraperitoneal injection of 0.2 ml sodium pentobarbital (50 mg ml^−1^). Mice were transcardially perfused with PBS followed by 4% paraformaldehyde in PBS. Tissue was removed and post-fixed for 24 h in 4% paraformaldehyde before being transferred to PBS or cryoprotected in 30% sucrose in PBS.

### Immunohistochemistry

Immunohistochemistry was performed as described previously^28^. Perfused post-mortem tissue was cryoprotected in 30% sucrose in PBS for 48 h before being embedded in cryomatrix (Tissue Tek O.C.T, Sakura Finetek) and freezing. Ten- or 20-micrometre-thick transverse sections of the spinal cord were cut on a cryostat (Leica), immediately mounted on glass slides and dried. Sections were blocked with 10% bovine serum albumin in PBS for 60 min. Then sections were incubated with the following primary antibody diluted in blocking solution at room temperature overnight: rabbit anti-GFAP (1:500, Dako Z0334), cFos (1:2,000 Synaptic Systems 226003), vGluT1 (1:1,000, Synaptic Systems 135302). Slides were washed four times with PBS before the secondary antibodies (Alexa Fluor Conjugated, ThermoFisher Scientific, USA) were applied for 90 min in blocking solution; donkey anti-rabbit Alexa Fluor 647 (1:1,000, A-31573), donkey anti-rat Alexa Fluor 647 (1:1,000, A-48272), donkey anti-goat Alexa Fluor 647 (1:1,000, A-21447). Slides were washed four times with PBS and then cover slipped with Mowiol. Immunofluorescence was imaged digitally using a slide scanner (Olympus VS-120 Slide scanner) or confocal microscope (Zeiss LSM880 + Airy fast module with ZEN 2 Black software (Zeiss)). Images were digitally processed using ImageJ (ImageJ NIH) software or Imaris (Bitplane, v.9.0.0).

### Pseudorabies tracing

The aim of the first set of experiments with the PRV Ba2017 was to optimize the timing required to achieve monosynaptic labelling of the brain after lumbar spinal cord infusions targeting the virus to Vsx2-positive neurons. Timepoints tested ranged from 1 day to 4 days using 12 h increments post-injection. Subsequently, a 3 day timepoint post-infusion was used for the remaining experiments. Post-mortem, brains were cut sagittally at 40 µm and directly mounted on glass slides. Every second section was imaged and imported into Neurolucida for brain reconstruction. Labelled neurons were counted per region.

### Fluorescence in situ hybridization

To verify the location of cell types, to uncover the identity of cells that provide input to or receive connections from SC^*Vsx2::Hoxa10*^ neurons, as well as to evaluate expression of activity-related genes in SC^*Vsx2::Hoxa10*^ neurons, we performed *in situ* hybridization of cell-type markers and activity-related genes using RNAscope (Advanced Cell Diagnostics). Lists of putative marker genes were obtained from snRNA-seq data, as described below, and cross-referenced against a list of validated probes designed and provided by Advanced Cell Diagnostics. In total, probes were obtained for the following genes: *Chat*, catalogue (cat.) no. 408731; *Maf*, cat. no. 412951; *Slc17a6*, cat. no. 319171; *Slc32a1*, cat. no. 319191; *Vsx2*, cat. no. 438341; *Pkd1l2*, cat. no. 520211; *Tac2*, cat. no. 446391. We then generated 12-µm cryosections from fixed-frozen spinal cords as previously described^66^ and performed fluorescence in situ hybridization for each probe according to the manufacturer’s instructions, using the RNAscope Fluorescent Multiplex Reagent Kit (cat. no. 323133) or HiPlex kit (cat. no. 324106).

### Tissue clearing

#### CLARITY

Samples were incubated in X-CLARITY hydrogel solution^67,68^ (Logos Biosystems) for 24 h at 4 °C with gentle shaking. Samples were degassed and polymerized using the X-CLARITY Polymerisation System (Logos Biosystems). Samples were washed in 0.001 M PBS for 5 min at room temperature then placed in the X-CLARITY Tissue Clearing System (Logos Biosystems), set to 1.5 A, 100 RPM, 37 °C, for 29 h. Clearing solution was made in house with 4% sodium dodecyl sulfate (SDS), 200 mM boric acid with dH_2_0, pH adjusted to 8.5. The samples were washed for at least 24 h at room temperature with gentle shaking in 0.1 M PBS solution containing 0.1% Triton X-100 to remove excess SDS. The samples were incubated in 40 g of Histodenz dissolved in 30 ml of 0.02 M PB, pH 7.5, 0.01% sodium azide (refractive index 1.465) for at least 24 h at room temperature with gentle shaking prior to imaging.

#### iDISCO+

Mice underwent a final EES^REHAB^ session and were perfused^69,70^ 60 min later with 0.1 M PBS followed by 4% PFA (in 0.1 M PBS). Samples were dissected and post-fixed in 4% PFA (in 0.1 M PBS) at 4 °C overnight and placed in 0.1 M PBS containing 0.03% sodium azide. Immunolabelling of the samples was performed by first pretreating with methanol in 2 ml Eppendorf tubes by dehydrating with a methanol/H_2_O series at 1 h each at room temperature with shaking at 60 RPM: 20%, 40%, 60%, 80% and 100%. This procedure was followed by 1 h washing with 100% methanol before chilling the samples at 4 °C. Samples were then incubated overnight with shaking in 66% dicholoromethane/33% methanol at room temperature. The samples were washed twice in 100% methanol with shaking at room temperature and then bleached in chilled fresh 5% H_2_O_2_ in methanol overnight at 4 °C. Samples were rehydrated with a methanol/H_2_O series: 80%, 60%, 40%, 20% and 0.1M PBS, each for 1 h at room temperature under shaking. Samples were washed for 1 h × 2 at room temperature in PTx.2 buffer (0.1 M PBS with 0.2% Triton X-100) under shaking. This was followed by an incubation in 2 ml of permeabilization solution (400 ml PTx.2, 11.5 g glycine, 100 ml DMSO for a total stock volume of 500 ml) for 2 days at 37 °C with shaking at 60 RPM. Samples were incubated in 2 ml of blocking solution (42 ml PTx.2, 3 ml of normal donkey serum, 5 ml of DMSO for a total stock volume of 50 ml) for 2 days at 37 °C with shaking. The samples were incubated for 7 days at 37 °C with shaking in primary antibody solution consisting of PTwH (0.1 M PBS, 2 ml Tween-20, 10 mg l^−1^ heparin, 5% dimethyl sulfoxide, 3% normal donkey serum), and cFos antibody (1:2,000, Synaptic Systems, 226003) for a total volume of 2 ml per sample. Samples were washed in PTwH for 24 h with shaking and incubated for 7 days at 37 °C with shaking in secondary antibody solution consisting of PTwH, 3% normal donkey serum and donkey anti-rabbit Alexa Fluor 647 (1:400, ThermoFisher Scientific) in a total volume of 2 ml per sample. Samples were washed in PTwH for 24 h with shaking at room temperature. Clearing of the samples was performed by first dehydrating the samples in a methanol/H_2_O series as follows: 20%, 40%, 60%, 80% and 100% twice each for 1 h with shaking at room temperature followed by a 3 h incubation with shaking in 66% dichloromethane/33% methanol at room temperature. Samples were incubated in 100% dichloromethane 15 min twice with shaking to wash residual methanol. Finally, samples were incubated in 100% dibenzyl ether without shaking for refractive index matching of the solution for at least 24 h prior to imaging.

#### uDISCO

uDISCO^71^ clearing of the mouse spinal cord was initiated by stepwise dehydration in increasing concentrations of *tert*-butanol diluted in dH_2_O with a total volume of 5 ml at 35 °C as follows: 30% *tert*-butanol overnight, 50% for 10 h, 70% overnight, 80% for 10 h, 90% overnight, 96% for 10 h and 100% overnight. The samples were then incubated in 5 ml of dichloromethane at room temperature for 70 min with shaking. This was then followed by incubation in BABB-D4 (BABB: 2:1 mixture of benzyl benzoate to benzyl alcohol; 4:1 mixture of BABB to diphenyl ether; 0.4% volumes vitamin E) for 24 h at room temperature shaking at 60 RPM prior to imaging.

### 3D imaging

Imaging of cleared tissue was performed using a customized MesoSPIM and CLARITY- optimized light-sheet microscope (COLM), as described^23,72^. A custom-built sample holder was used to secure the central nervous system in a chamber filled with RIMS. Samples were imaged using either a 1.25× or 2.5× objective at the mesoSPIM^72^ and a 4× or 10× objective at the COLM^23^ with one or two light sheets illuminating the sample from the left and right sides. The voxel resolution in the *x*, *y* and *z* directions was 5.3 µm × 5.3 µm × 5 µm for the 1.25× acquisition and 2.6 µm × 2.6 µm × 3 µm for the 2.5× acquisition. The voxel resolution of the COLM was 1.4 µm × 1.4 µm by 5 um for the 4× and 0.59 µm × 0.59 µm × 3 µm for the 10× acquisition. Images were generated as 16-bit TIFF files and then stitched using Arivis Vision4D (Arivis). 3D reconstructions and optical sections of raw images were generated using Imaris (Bitplane, V.9.0.2) software.

### cFos quantification

cFos positive neurons of cleared samples were quantified using Arivis Vision4D (Arivis). After defining a region of interest around the grey matter, each sample was subjected to a custom-made pipeline. We applied morphology, denoising, and normalization filters to enhance the signal of bright objects and homogenized the background. Threshold-based segmentation of the cFos signal was applied within predefined 3D regions to quantify the total number of cFos positive cells. Image analysis parameters were kept constant among all samples.

### Neuron-specific recordings and analysis

AAV5-Syn-flex-Chrimson was infused in the lumbar spinal cord of *Vsx2*^*cre*^ mice at least four weeks prior to terminal experiments. Mice were anaesthetized with ketamine/xylazine. Ketamine maintenance doses were then administered as needed. Four-shank, multi-site electrode arrays (NeuroNexus A4x16-Poly2-5 mm-23s-200-177) were lowered into the spinal cord to a depth of 800 µm, with shanks arranged longitudinally at 350 µm from midline. Signals were recorded with a NeuroNexus Smartbox Pro using a common average reference and while applying 50 Hz notch and 450–5,000 Hz bandpass filters. Stimulation was controlled with a Multi-Channel Systems STG 4004 and MC_Stimulus II software. ChrimsonR-expressing neurons were identified using optogenetic stimulation. Ten pulse trains of 1-ms pulses were delivered at 20 Hz from a 635 nm laser (LaserGlow Technologies LRD-0635-PFR-00100-03). Laser light was delivered to the surface of the spinal cord through a fibre optic cable attached to 400 µm, 0.39 NA cannula with a 5 mm tip (Thorlabs). Optical power was set to 2.35 mW at the tip. Electrical stimulation (EES and reticular formation) consisted of 200*-*µs pulses delivered every 5 s. EES was delivered with a micro fork probe (Inomed, 45 mm straight, item no. 522610) positioned along the midline just caudal to the recording array. Stimulation of the reticular formation was delivered with a platinum/iridium bipolar concentric electrode (Microprobes PI-SNE-100) placed at −5.8 mm caudal and 0.3 lateral from Bregma, 5.6 mm deep from the brain surface. Spike sorting was performed with SpyKING CIRCUS v.1.0.7^73^. The median-based average electrical stimulation artefacts for each channel were subtracted from the recordings prior to sorting. Due to the size and variability of the artefacts, periods containing residual stimulation artefacts were not sorted (–1.0 to +1.5 ms and +2.5 ms around stimulus onset for EES and reticular formation stimulation onset, respectively). Sorting results were manually curated using Phy (https://github.com/cortex-lab/phy). Single unit clusters were selected for analysis based on their biphasic waveforms and template amplitudes above 50 µV, as well as strong refractory period dips in their spike autocorrelograms. Similar clusters were merged according to the Phy manual clustering guide. ChrimsonR-expressing, putative SC^*Vsx2::Hoxa10*^ neurons were identified based on their low-latency and lowjitter responses to light pulses. Specifically, a one-sided Wilcoxon signed-rank test was used to compare the instantaneous firing rate of units 10 ms before stimulus onset and 6 ms after. Those neurons with a post-stimulus onset firing rate increase of *P* value less than 0.001 and response jitter (standard deviation of latency) less than 2 ms were considered to be directly activated and therefore SC^*Vsx2::Hoxa10*^ neurons that displayed early responses to electrical stimulation (EES or reticular formation), indicative of putative monosynaptic inputs, were identified using the same firing rate test in addition to a jitter of less than 1.2 ms and minimum spike probability in the first 6 ms after stimulation greater than 20%. For stimulation of the reticular formation, the post-stimulus onset quantification window was increased to 7 ms to account for conduction time from the brainstem to the spinal cord^74^.

### Fabrication of optoelectronic implants integrating microLEDs and EES

The fabrication of the micro-LED array was based on a previously validated technological design^40^ that we adapted to incorporate electrodes to deliver EES, as illustrated in Extended Data Fig. 15a. A 4-inch silicon wafer coated with Ti/Al (25/100 nm) was spin-coated with a 3 mm thick polyimide layer (PI2610, HD Microsystems), followed by a curing process of 2 h at 300 °C in a N_2_ oven. A Ti/Au/Ti metal layer (25/250/25 nm) was sputtered after O_2_ plasma surface activation (AC450, Alliance Concept). Wet etching for Au and reactive ion etching (RIE) for Ti patterned the metal layer after the photolithography (AZ1512, MicroChemicals). A polyimide layer (2-µm thick) was then spin-coated and cured to encapsulate the patterned metallization layer. Photolithography (AZ9620, MicroChemicals) and RIE etching patterned the polyimide-metal-polyimide stack. Next, A 20-nm-thick SiO_2_ layer was sputtered and followed by the spin-coating of a 15*-*µm-thick polydimethylsiloxane (PDMS) (Sylgard 184, Dow Corning) after O_2_ plasma surface activation. RIE etching of the PDMS layer after the photolithography (AZ40XT, MicroChemicals) exposed openings for the micro-LED integration sites and electrical stimulation sites. For the micro-LED integration, the solder paste (diameter 50 mm, SMDLTLFP10T5, Chipquik) and blue microLEDs (TR2227, Cree) were deposited onto the contact pads using a pick-and-place equipment (JFP Microtechnic). The microLEDs were bonded through the solder paste reflow at 165 °C and the exposed connection areas around microLEDs were drop-cast with a 14 wt% solution of polyisobutylene (PIB, Oppanol, BASF) in cyclohexane (Sigma-Aldrich). After solvent evaporation (10 min at 55 °C), the device was cured for 4 h at room temperature. Next, a 50 wt % PDMS-phosphor composite (HTR620, PhosphorTech) was deposited over the PIB-encapsulated blue microLEDs via pneumatic printing to activate red-shifted opsins. Electrical stimulation sites were coated after O_2_ plasma surface activation with a platinum-silicone composite comprised of 100 µg of platinum microparticle powder (Goodfellow) mixed into a 110 ml PDMS/cyclohexane solution (200 mg/500 ml, Dow Corning, Sigma-Aldrich). The mixture turned into a paste after the cyclohexane evaporation, and the paste was placed onto a laser-cut PET mask to selectively fill openings via screen printing. After the PET mask removal, the device was cured overnight at room temperature, followed by 4 h at 55 °C. A 50*-*µm-thick PDMS layer was manually spread on the surface of the implant except for the conductive composite sites (~70 mm) for ease of handling during the surgical process. Stainless steel wires from a circular connector (A71914/A71915, Omnetics) were soldered onto the implant pads and sealed with silicone (734, Dow Corning). As a last step, the implant was released from the wafer through anodic dissolution of the Al layer at 1.2 V bias in a NaCl-saturated deionized water.

### Implantation of the optoelectronic implant

Procedures have been described in detail previously^40^. In brief, the interlaminar spaces between vertebrae T12/T13 and L2/L3 were dissected to expose the exit and entry points, respectively, for the implant. A 6-0 ethilon suture (MPE697H, Ethicon EMEA) was passed through the epidural space from T12/T13 to L2/L3, then through the silicone loop of the implant and back through the epidural space. The implant was slid over the spinal cord by gently pulling both ends of the suture rostrally. The connector of the implant was fixed by suturing paraspinal muscles across the connector. The percutaneous connector (16-pin connector, A79112-001, Omnetics Connector Corporation) and wires were routed subcutaneously to the head. Three screws (part no. AMS120/5B-25, Antrin miniature specialties) were inserted into the skull, surrounding the percutaneous connector. Fresh dental cement was then poured around the screws and connector, and held in place until cured. Photostimulation protocols for inhibition of SC^*Vsx2::Hoxa10*^ consisted of 20 ms pulses at 50 Hz (100% duty cycle).

### Statistical procedures

Behavioural assays were replicated 3 to 10 times depending on the experiments and averaged per animal. Statistical analysis was performed in R (version 3.6.3). Two-sided paired or independent *t*-tests, one-way ANOVA or two-way repeated measures ANOVA were used as appropriate, followed by post hoc significance testing. Alpha was set as 0.05. Non-parametric Mann–Whitney or Wilcoxon signed-rank tests were used when comparisons involved fewer than five mice. Representative experiments such as histological micrographs were repeated in a minimum of four mice.

### Single-nucleus RNA sequencing

We performed single-nucleus dissociation of the mouse lumbar spinal cord according to our previously described procedures^9,12^. Following euthanasia by isoflurane inhalation and cervical dislocation, the lumbar spinal cord site was immediately dissected and frozen on dry ice. We denounced spinal cords in 500 µl sucrose buffer (0.32 M sucrose, 10 mM HEPES pH 8.0, 5 mM CaCl_2_, 3 mM magnesium acetate, 0.1 mM EDTA, 1 mM DTT) and 0.1% Triton X-100 with the Kontes Dounce Tissue Grinder. Two millilitres of sucrose buffer was then added and filtered through a micrometre cell strainer. The lysate was centrifuged at 3,200 *g* for 10 min at 4 °C. The supernatant was decanted, and 3 ml of sucrose buffer was added to the pellet and incubated for 1 min. The pellet was homogenized using an Ultra-Turrax and 12.5 ml density buffer (1 M sucrose, 10 mM HEPES pH 8.0, 3 mM Mg acetate, 1 mM DTT) was added below the nuclei layer. The tube was centrifuged at 3,200 *g* at 4 °C and supernatant poured off. Nuclei on the bottom half of the tube wall were collected with 100 µl PBS with 0.04% BSA and 0.2 U µl^−1^ RNase inhibitor. Resuspended nuclei were filtered through a 30*-*µm strainer, and adjusted to 1,000 nuclei per µl.

### Library preparation

We carried out snRNA-seq library preparation using the 10x Genomics Chromium Single Cell Kit version 2. The nuclei suspension was added to the Chromium RT mix to achieve loading numbers of 5,000. For downstream cDNA synthesis (13 PCR cycles), library preparation and sequencing, the manufacturer’s instructions were followed.

### Read alignment

Reads were aligned to the most recent Ensembl release (GRCm38.93) using Cell Ranger, and a matrix of UMI counts, including both intronic and exonic reads, was obtained using velocyto^75^. Seurat^30^ was used to calculate quality control metrics for each cell barcode, including the number of genes detected, number of UMIs, and proportion of reads aligned to mitochondrial genes. Low-quality cells were filtered by removing cells expressing less than 200 genes or with more than 5% mitochondrial reads. Genes expressed in less than three cells were likewise removed, yielding a count matrix consisting of 22,806 genes in 81,657 cells.

### Clustering and integration

Prior to clustering analysis, we first performed batch effect correction and data integration across the two different experimental conditions as previously described^30^. Gene-expression data was normalized using regularized negative binomial models^76^, then integrated across batches using the data integration workflow within Seurat. The normalized and integrated gene expression matrices were then subjected to clustering to identify cell types in the integrated dataset, again using the default Seurat workflow. Cell types were manually annotated on the basis of marker gene expression, guided by previous studies of the mouse spinal cord^9,12,31,32^.

### RNA velocity

RNA velocity was calculated using the velocyto package^75^. Velocyto estimates cell velocities from their spliced and unspliced mRNA content. We generated the annotated spliced and unspliced reads using the run10x function of the Velocyto command line tool. We then calculated gene-relative velocity using *k*-nearest neighbour pooling with *k* = 10 (default), quantile = 0.1 (default). For snRNA-seq, we set emat to 0.006 and nmat to 0.004. For spatial transcriptomics, we set emat to 0.01 and nmat to 0.01.

### Spatial transcriptomics

The lumbar spinal cords of mice were embedded in OCT and cryosections were generated at 10 µm at –20 °C. Sections were immediately placed on chilled Visium Tissue Optimization Slides (cat. no. 1000193, 10x Genomics) or Visium Spatial Gene Expression Slides (cat. no. 1000184, 10x Genomics). Tissue sections were then fixed in chilled methanol and stained according to the Visium Spatial Gene Expression User Guide (cat. no. CG000239 Rev A, 10x Genomics) or Visium Spatial Tissue Optimization User Guide (cat. no. CG000238 Rev A, 10x Genomics). For gene-expression samples, tissue was permeabilized for 12 min, which was selected as the optimal time based on tissue-optimization time-course experiments. Bright-field histology images were taken using a 10× objective on a slide scanner (Olympus VS-120 Slide scanner). For tissue-optimization experiments, fluorescent images were taken with a TRITC filter using a 10× objective and 400 ms exposure time.

Spatial transcriptomics libraries were prepared according to the Visium Spatial Gene Expression User Guide. They were then clustered at 270 pM on a paired-end HiSeq4000 flow cell and sequenced on a HiSeq4000 System (Illumina) at a sequencing depth of approximately 140–180 million reads per sample. Sequencing was performed using the following read protocol: read 1: 28 cycles; i7 index read: 8 cycles; i5 index read: 8 cycles; and read 2: 98 cycles.

Raw FASTQ files and histology images were processed using the Space Ranger software (version 1.0.0). Reads were aligned to the most recent Ensembl release (GRCm38.93), and a matrix of UMI counts, including both intronic and exonic reads, was obtained using velocyto^75^. Seurat^30^ was used to calculate quality control metrics for each cell barcode, including the number of genes detected, number of UMIs, and proportion of reads aligned to mitochondrial genes. Low-quality barcodes were filtered by removing cells expressing less than 5,000 UMIs. Genes expressed in less than 3 barcodes were likewise removed, yielding a UMI count matrix consisting of 22,127 genes and 9,755 barcodes across 61 spinal cord sections from 12 mice from four experimental conditions. To align all sections to a common coordinate space, we implemented a custom image analysis pipeline that includes preprocessing, registration and combination of histological images from different sections. In brief, we implemented all preprocessing in Fiji, and all registration procedures in R, using the image analysis package imageR, and medical image registration package RNiftyReg. Images were aligned to the L3 spinal cord segment from a standard histological atlas. Regions were assigned on the basis of their location within the cytoarchitecture of the spinal segment.

### Cell type deconvolution

To integrate our spatial and single-nucleus transcriptomes, we used robust cell type decomposition^77^ (RCTD) to localize each neuronal subpopulation inferred from snRNA-seq data within the mouse spinal cord. In brief, RCTD deconvolves each spatial barcode into a mixture of one or more neuronal subpopulations, while accounting for technical differences between single-nucleus and spatial transcriptomes. RCTD was run with doublet mode disabled, allowing each barcode to potentially contain more than two cell types, to produce a matrix of 9,755 spatial barcodes by 36 neuronal subpopulations. The cells in this matrix represented the neuronal subpopulation or subpopulations inferred at each spatial barcode. We observed spatial distributions that were largely compatible with existing knowledge about the locations of each neuronal subtype within the spinal cord, notwithstanding some minor discrepancies, which can likely be attributed to the relatively large regions captured by each spatial barcode, the depth of our snRNA-seq data, and the higher degree of transcriptional similarity between ventral neuronal subpopulations in the spinal cord. We visualized the spatial distribution of neuronal subpopulations by plotting the scores assigned for each subpopulation within the common coordinate system of the registered spinal cord. We recovered smoothed patterns of spatial deconvolution with two-dimensional locally weighted regression, as described by the authors of RCTD^77^. In addition, we computed the mean RCTD score for each neuronal subpopulation in each of the five spinal cord regions assigned by the Allen Brain Atlas.

### Differential expression

To identify genes differentially expressed in SC^*Vsx2::Hoxa10*^ neurons, we performed differential expression testing within each comparison using negative binomial generalized linear mixed models (GLMMs), as previously described^78^ and as implemented in the Libra R package, available at https://github.com/neurorestore/Libra. We incorporated library size factors as an offset term, and calculated statistical significance using a likelihood ratio test against a reduced model. We focused our analysis on VE-1 neurons, as these were prioritized by Augur with the highest median AUC across all six comparisons. To further dissect the significance of the observed transcriptional changes in response to perturbations on different timescales, we then divided the six comparisons into groups of acute and chronic perturbations. The chronic perturbations consisted of EES^REHAB^ versus SCI and EES^REHAB^^→EES::walking^ versus SCI^→EES::walking^; the remaining perturbations were considered to be acute perturbations. We tested for enrichment of immediate early genes, using a list of genes manually curated by Hrvatin et al.^79^, by applying fgsea^80^ to the signed −log_10_ *P* values estimated by the GLMMs, and compared the normalized enrichment scores estimated by fgsea between acute and chronic perturbations. To identify genes specifically upregulated in chronic perturbations, we performed a one-sided *t*-test on the coefficients estimated by the GLMMs, limiting our analysis to genes for which the GLMMs could be fit in all six comparisons.

### Cell type prioritization with Augur

To identify neuronal subpopulations activated by key therapeutic features of EES^REHAB^, we developed a machine learning method that we named Augur^12,13^. The key assumption underlying Augur is that cell types undergoing a profound response to a perturbation should become more separable, within the highly multidimensional space of gene expression, than less affected cell types. To quantify this separability, we framed this problem as a classification task. In brief, Augur withholds a proportion of sample labels, then trains a random forest classifier to predict the condition from which each cell was obtained (for instance, diseased or healthy tissue). The accuracy with which this prediction can be made from single-cell gene expression measurements is then evaluated in cross-validation, and quantified using the area under the receiver operating characteristic curve (AUC). This process is repeated separately for each cell type, such that the AUC provides a quantitative measure of separability that can be used to rank cell types based on the relative magnitude of their response to an arbitrary perturbation. We refer to this process as cell type prioritization.

To identify the neuronal subpopulations engaged by EES^REHAB^, we applied Augur to six key comparisons involving the eight experimental groups. Five of these six comparisons involved acute perturbations on the timescale of transcription (minutes to hours), raising a risk that the total transcriptional output of each cell may not yet fully reflect the impact of the perturbation at the time of snRNA-seq^12^. Consequently, for these five comparisons, we applied Augur to a matrix of RNA velocity^75^, as previously described^12,13,75^. For the remaining comparison (EES^REHAB^ vs SCI), Augur was applied directly to the UMI count matrix. Augur was run with default parameters for all comparisons. To evaluate the robustness of cell type prioritizations to the resolution at which neuronal subpopulations were defined in the snRNA-seq data, we applied Augur at various clustering resolutions, and visualized the resulting cell type prioritizations both on a hierarchical clustering tree^81^ of neuron subpopulations and as a progression of UMAPs.

### Spatial prioritization with Magellan

To enable spatial prioritization of the perturbation response within complex tissues, we developed Magellan. Magellan builds on the concept of transcriptional separability that provides a basis for cell type prioritization in Augur. However, in spatial transcriptomics data, the analytical level of interest is not necessarily a cell type, but rather a location within a two- or three-dimensional tissue. To approach the data at this level, we sought to evaluate the transcriptional separability between barcodes from two experimental conditions at each point within a common coordinate system. We reasoned that we could achieve this by evaluating the separability of barcodes from each condition within small, overlapping tiles, layered across the spatial coordinate system. In brief, for each barcode in a spatial transcriptomics dataset, Magellan selects the *k*-nearest neighbours from each experimental condition within common coordinate space, where *k* is set to 20 by default. Then, Magellan withholds the experimental condition labels for a proportion of these neighbours, and trains a random forest classifier to predict the experimental condition given the remaining barcodes as input. The accuracy of these predictions is evaluated in the withheld barcodes, and the process is repeated in three-fold cross-validation. As in Augur, the accuracy is quantified using the AUC. The cross-validation is repeated several times (by default, 50 times) in order to converge at a robust estimate of the AUC. The entire procedure is repeated for each barcode in the dataset, providing a spatial map of the AUC over the coordinate system of the spatial transcriptomes.

We then generated simulated data to validate Magellan. We devised a sequence of spatial patterns that we reasoned would capture key desiderata of a spatial prioritization method. Our premise was that an ideal method would be capable of detecting both sharp borders and smooth gradients, and would be capable of resolving quantitative differences between different aspects of a multifaceted perturbation response. We used Splatter^82^ to simulate spatial transcriptomics data for six patterns that captured this premise. Simulation parameters were estimated from our own spatial transcriptomics dataset using the splatEstimate function. A total of 5,000 barcodes were simulated for each pattern, and arranged within the common coordinate system of the mouse lumbar spinal cord. A fixed proportion of 10% of all genes in the dataset were set to be differentially expressed between the two simulated conditions (using the de.prob parameter), with the intensity of the differential expression varying according to the simulated spatial pattern (controlled with the de.facLoc parameter). Magellan was applied to each simulated dataset with default parameters, with two exceptions. First, cross-validation was repeated 100 times for each barcode in order to estimate the number of repeated cross-validation folds required to obtain a robust estimate, which was achieved by comparing the AUC assigned to each barcode in the first 50 samples to the second 50 samples. Second, to evaluate the effect of the parameter *k*, we ran Magellan with *k* = 20, 50 or 100 neighbours for each spatial barcode.

We implemented Magellan as an R package, available from https://github.com/neurorestore/Magellan. Magellan builds on Augur by reusing its procedures for feature selection, classification, and cross-validation. However, unlike Augur, Magellan requires as input a spatial transcriptomics dataset in which each barcode is associated with a two-dimensional coordinate and an experimental condition.

To enable spatial prioritization in our own dataset, we applied Magellan to four key comparisons between four experimental conditions. Barcodes from the white matter were excluded, and two low-quality sections excluded (Slide1_4_B1_M3_6wNT, Slide1_2_B3_M10_6wNT). In view of the left-right symmetry of the spinal cord, we replaced the coordinate of each barcode on the *x*-axis with its absolute value in order to mitigate artefacts introduced by the registration procedure. Magellan was run with default parameters (*k* = 20) and with *k* = 50 or 100 in order to illustrate the effect of spatial resolution on our results. We used an identical local regression procedure to that described above for spatial deconvolution with RCTD to visualize smoothed spatial prioritizations^77^.

### Embedding single-nucleus transcriptomes in space

To corroborate the spatial prioritizations assigned by Magellan, we leveraged Tangram^36^ to embed single-nucleus transcriptomes within the common coordinate system of the mouse spinal cord. We aligned single-cell barcodes from each of the four matching experimental conditions to the corresponding spatial transcriptomics data, using 100 marker genes and training the model for 100 epochs. Spatial barcodes from the white matter were excluded from this analysis. This procedure assigned a spatial coordinate to each single-nucleus transcriptome. We then correlated the AUCs assigned by Augur to each single-nucleus transcriptome to the AUCs assigned by Magellan at the matching spatial coordinates. Finally, to approximate the degree of concordance between cell type and spatial prioritizations, we computed the mean AUC for all spatial barcodes overlapping with single-nucleus transcriptomes of a given cell type.

### Computer simulations

We performed computer simulations on a previously validated spiking neural network model^37,38^, reproducing the proprioceptive feedback circuits associated with a pair of antagonist muscles in the lumbar spinal cord of rodents. Computer simulations were performed in Python 2.7 using the NEURON simulation environment^83^.

The spiking neural network includes populations of group Ia and group II afferent fibres, Ia inhibitory interneurons, group II excitatory interneurons, and pools of alpha motor neurons. The number of cells, the number and the strength of the synapses contacting the different populations of neurons, and the characteristics of the cell models are described in our previous work^37,38^.

A validated finite element model of EES of the lumbar spinal cord^37,38^ was used to estimate the proportion of afferent and efferent fibres recruited at a given stimulation amplitude.

### Reporting summary

Further information on research design is available in the Nature Research Reporting Summary linked to this article.

## Online content

Any methods, additional references, Nature Research reporting summaries, source data, extended data, supplementary information, acknowledgements, peer review information; details of author contributions and competing interests; and statements of data and code availability are available at 10.1038/s41586-022-05385-7.

## Supplementary information

Supplementary Information This file contains a description of the clinical trial STIMO and Supplementary Tables 1–3.

Reporting Summary

Supplementary Video 1 EES^REHAB^ restores walking in 9 humans with chronic spinal cord injury.

Supplementary Video 2 Mouse model replicating the key technological and therapeutic features of EES^REHAB^ in humans.

Supplementary Video 3 Molecular cartography of recovery from paralysis.

Supplementary Video 4 SC^*Vsx2::Hoxa10*^ neurons are not necessary for walking in uninjured mice.

Supplementary Video 5 SC^*Vsx2::Hoxa10*^ neurons are necessary and sufficient to regain walking after paralysis.

## Data Availability

Raw sequencing data and count matrices have been deposited to the Gene Expression Omnibus under accessions GSE184370 (snRNA-seq) and GSE184369 (spatial transcriptomics). Source data are provided with this paper.

## Source data

Source Data Fig. 1
Source Data Fig. 3
Source Data Fig. 4
Source Data Extended Data Fig. 1
Source Data Extended Data Fig. 2
Source Data Extended Data Fig. 12
Source Data Extended Data Fig. 13
Source Data Extended Data Fig. 14
Source Data Extended Data Fig. 15

## Code availability

Augur is available from GitHub (https://github.com/neurorestore/Augur). Magellan is available from GitHub (https://github.com/neurorestore/Magellan) and as Supplementary Software 1.
