# Supplementary material for: The neurons that restore walking after paralysis
Source: Nature. 2022 Nov 9;611(7936):540–7. doi: 10.1038/s41586-022-05385-7 (PMC9668750; doi:10.1038/s41586-022-05385-7)
Supplement: Supplementary file 1 — This file contains a description of the clinical trial STIMO and Supplementary Tables 1–3. [file 41586_2022_5385_MOESM1_ESM.pdf]

nature portfolio

https://doi.org/10.1038/s41586-022-05385-7

Supplementary information

The neurons that restore walking after paralysis

In the format provided by the authors and unedited

nature portfolio

https://doi.org/10.1038/s41586-022-05385-7

Supplementary information

The neurons that restore walking after paralysis

In the format provided by the authors and unedited

Supplementary Information guide

Description of the clinical trial STIMO 2

Supplementary Tables and Legends.. 5

Description of the clinical trial STIMO
Clinicaltrials.gov: NCT02936453

Study context. All experiments were carried out as part of the ongoing clinical feasibility study STIMO ("Stimulation Movement Overground"), which investigates the effects of spatiotemporal EES combined with weight-supported overground locomotor training on the recovery of motor function after SCI. This study was approved by the Swiss ethical authorities (Swissethics protocol number 04/2014 ProjectID: PB_2016-00886, Swissmedic protocol 2016-MD-0002) and was conducted in accordance with the Declaration of Helsinki. All participants signed a written informed consent prior to their participation. More information is provided at clinicaltrials.gov (NCT02936453). All surgical and experimental procedures were performed at the Lausanne University Hospital (CHUV) and have been previously described in detail[19].

Study Objectives. Primary objective

Address the feasibility in terms of efficacy and safety of overground, robot-assisted neurorehabilitation in combination with spinal EES to reduce the need of assistance required to walk and to increase the speed of walking, i.e., facilitate motor control, in chronic, SCI patients.

Secondary objective

Address the efficacy of overground, robot-assisted neurorehabilitation in combination with spinal EES to improve independence in activities of daily living and to improve endurance during walking, i.e., facilitate motor control, in chronic, SCI patients.

Study Endpoints. Primary endpoints

The overground, robot-assisted neurorehabilitation in combination with spinal EES will result in less assistance required to walk and faster speed of walking. This will be calculated within each individual and across the group (12 patients). *Chosen measures: WISCI II Score, 10-Meter Walk Test, Weight Bearing Capacity (WBC).*

Secondary endpoints

The overground, robot-assisted neurorehabilitation in combination with spinal EES will result in more independence in activities of daily living and an improved endurance during standing and walking. This will be calculated within each individual and across the group (12 patients). *Chosen measures: SCIM III Score, 6-Minute Walk Test.*

Inclusion Criteria. Patients fulfilling all of the following inclusion criteria may be enrolled in the study:

- Age 18-65 (women or men)
- Sensorimotor or motor complete and incomplete SCI graded as AIS A, B, C & D
- Level of lesion: T10 and above, based on AIS level determination by the PI, with preservation of conus function

- The intact distance between the cone and the lesion must be at least 60 mm.
- Focal spinal cord disorder caused by either trauma or epidural, subdural or intramedullary bleeding
- Minimum 12 months post-injury
- Completed in-patient rehabilitation program
- For ASIA C and D, able to stand with walker or 2 crutches
- Stable medical, physical and psychological condition as considered by Investigators
- Able to understand and interact with the study team in French or English
- Adequate care-giver support and access to appropriate medical care in patient's home community
- Agree to comply in good faith with all conditions of the study and to attend all required study training and visit
- Must participate in two training sessions before eligibility is confirmed
- Must provide and sign Informed Consent prior to any study related procedures

***Exclusion Criteria*.**

The presence of any one of the following exclusion criteria will lead to exclusion of the subject:

- Limitation of walking function based on accompanying (CNS) disorders (systemic malignant disorders, cardiovascular disorders restricting physical training, peripheral nerve disorders)
- History of significant autonomic dysreflexia
- Cognitive/brain damage
- Epilepsy
- Patient who has spinal canal stenosis
- Patient who uses an intrathecal Baclofen pump.
- Patient who has any active implanted cardiac device such as pacemaker or defibrillator.
- Patient who has any indication that would require diathermy.
- Patient who has any indication that would require MRI.
- Patients that have an increased risk for defibrillation
- Severe joint contractures disabling or restricting lower limb movements.
- Haematological disorders with increased risk for surgical interventions (increased risk of haemorrhagic events).
- Participation in another locomotor training study.
- Congenital or acquired lower limb abnormalities (affection of joints and bone).
- Women who are pregnant (pregnancy test obligatory for woman of childbearing potential) or breast feeding or not willing to take contraception.
- Known or suspected non-compliance, drug or alcohol abuse.
- Spinal cord lesion due to either a neurodegenerative disease or a tumour.
- Patient has other anatomic or co-morbid conditions that, in the investigator's opinion, could limit the patient's ability to participate in

the study or to comply with follow-up requirements, or impact the scientific soundness of the study results.

- Patient is unlikely to survive the protocol follow-up period of 12 months.

Study timeline.

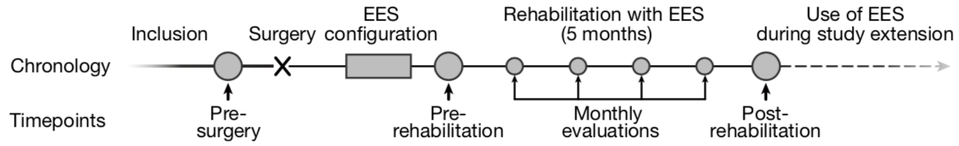

The study involves assessments before surgery, the surgical implantation of the neurostimulation system, a one-month period during which EES protocols are configured, and a five-month rehabilitation period with physiotherapists taking place four to five times per week for one to three hours. The rehabilitation program is personalized based on the current capacities and improvements displayed by each participant. At the end of the rehabilitation period, the participants are given the opportunity to be enrolled in a study extension phase during which they can continue using the neurostimulation system at home. Participants enrolled in this extension phase are followed-up on a regular basis by the study team for up to six years.

Supplementary Tables and Legends

<table>
<tr><th>Participants</th><th colspan="2">BT001</th><th colspan="2">DM002</th><th colspan="2">GO004</th><th colspan="2">ST006</th><th colspan="2">MB007</th><th colspan="2">HT008</th><th colspan="2">GF009</th><th colspan="2">GJ010</th><th colspan="2">MR012</th></tr>
<tr><td>Gender</td><td colspan="2">m</td><td colspan="2">m</td><td colspan="2">m</td><td colspan="2">m</td><td colspan="2">m</td><td colspan="2">m</td><td colspan="2">m</td><td colspan="2">m</td><td colspan="2">m</td></tr>
<tr><td>Age at study enrolment (y)</td><td colspan="2">53</td><td colspan="2">28</td><td colspan="2">34</td><td colspan="2">47</td><td colspan="2">23</td><td colspan="2">32</td><td colspan="2">32</td><td colspan="2">41</td><td colspan="2">29</td></tr>
<tr><td>Years after SCI at study enrolment</td><td colspan="2">3 years and 2 months</td><td colspan="2">5 years and 11 months</td><td colspan="2">5 years and 5 months</td><td colspan="2">4 years and 3 months</td><td colspan="2">3 years and 8 months</td><td colspan="2">14 years and 3 months</td><td colspan="2">8 years and 11 months</td><td colspan="2">1 year and 3 months</td><td colspan="2">2 years and 10 months</td></tr>
<tr><td>Assessment at study enrolment (Pre)
and after rehabilitation period (Post)</td><td>Pre</td><td>Post</td><td>Pre</td><td>Post</td><td>Pre</td><td>Post</td><td>Pre</td><td>Post</td><td>Pre</td><td>Post</td><td>Pre</td><td>Post</td><td>Pre</td><td>Post</td><td>Pre</td><td>Post</td><td>Pre</td><td>Post</td></tr>
<tr><td>American Spinal Injury Association Impairment Scale (AIS)</td><td>D</td><td>D</td><td>C</td><td>D</td><td>C</td><td>D</td><td>C</td><td>C</td><td>C</td><td>C</td><td>C</td><td>D</td><td>A</td><td>A</td><td>A</td><td>A</td><td>B</td><td>C</td></tr>
<tr><td>Neurological level of injury</td><td></td><td></td><td></td><td></td><td></td><td></td><td></td><td></td><td></td><td></td><td></td><td></td><td>T4</td><td>T3</td><td>T3</td><td>T3</td><td>T7</td><td>T7</td></tr>
<tr><td>Lower Extremity Motor Score</td><td></td><td></td><td></td><td></td><td></td><td></td><td></td><td></td><td></td><td></td><td></td><td></td><td></td><td></td><td></td><td></td><td></td><td></td></tr>
<tr><td>L2, hip flexors (right | left)</td><td>2 | 3</td><td>4 | 4</td><td>2 | 0</td><td>4 | 2</td><td>2 | 2</td><td>3 | 2</td><td>0 | 0</td><td>0 | 1</td><td>0 | 0</td><td>1 | 1</td><td>0 | 2</td><td>2 | 4</td><td>0 | 0</td><td>0 | 0</td><td>0 | 0</td><td>0 | 0</td><td>0 | 0</td><td>1 | 2</td></tr>
<tr><td>L3, knee extensors (right | left)</td><td>4 | 4</td><td>4 | 4</td><td>2 | 0</td><td>4 | 3</td><td>4 | 4</td><td>4 | 4</td><td>0 | 0</td><td>1 | 0</td><td>0 | 0</td><td>0 | 0</td><td>1 | 2</td><td>4 | 4</td><td>0 | 0</td><td>0 | 0</td><td>0 | 0</td><td>0 | 0</td><td>0 | 0</td><td>1 | 1</td></tr>
<tr><td>L4, ankle dorsiflexors (right | left)</td><td>2 | 2</td><td>3 | 2</td><td>4 | 0</td><td>4 | 1</td><td>3 | 3</td><td>4 | 4</td><td>0 | 0</td><td>0 | 1</td><td>0 | 0</td><td>0 | 0</td><td>0 | 1</td><td>2 | 4</td><td>0 | 0</td><td>0 | 0</td><td>0 | 0</td><td>0 | 0</td><td>0 | 0</td><td>0 | 1</td></tr>
<tr><td>L5, long toe extensors (right | left)</td><td>4 | 4</td><td>0 | 0</td><td>4 | 0</td><td>4 | 2</td><td>1 | 1</td><td>2 | 4</td><td>0 | 0</td><td>0 | 1</td><td>0 | 0</td><td>3 | 3</td><td>1 | 4</td><td>0 | 4</td><td>0 | 0</td><td>0 | 0</td><td>0 | 0</td><td>0 | 0</td><td>0 | 0</td><td>0 | 0</td></tr>
<tr><td>S1, ankle plantar flexors (right | left)
(max. 5 per side)</td><td>2 | 2</td><td>4 | 4</td><td>2 | 0</td><td>4 | 2</td><td>4 | 4</td><td>5 | 4</td><td>0 | 0</td><td>0 | 0</td><td>0 | 0</td><td>0 | 0</td><td>0 | 1</td><td>0 | 2</td><td>0 | 0</td><td>0 | 0</td><td>0 | 0</td><td>0 | 0</td><td>0 | 0</td><td>0 | 0</td></tr>
<tr><td>Total (max. 25 | 25)</td><td>14 | 15</td><td>4 | 5</td><td>14 | 0</td><td>20 | 0</td><td>14 | 14</td><td>18 | 18</td><td>0 | 0</td><td>1 | 3</td><td>0 | 0</td><td>4 | 4</td><td>2 | 10</td><td>8 | 18</td><td>0 | 0</td><td>0 | 0</td><td>0 | 0</td><td>0 | 0</td><td>0 | 0</td><td>2 | 4</td></tr>
<tr><td>Deep anal pressure (DAP)</td><td></td><td></td><td></td><td></td><td></td><td></td><td></td><td></td><td></td><td></td><td></td><td></td><td>No</td><td>No</td><td>No</td><td>No</td><td>Yes</td><td>Yes</td></tr>
<tr><td>Voluntary anal contraction (VAC)</td><td></td><td></td><td></td><td></td><td></td><td></td><td></td><td></td><td></td><td></td><td></td><td></td><td>No</td><td>No</td><td>No</td><td>No</td><td>No</td><td>No</td></tr>
<tr><td>Light-Touch Sensory score
L1-S2 dermatomes subscore (right | left)</td><td>9 | 9</td><td>9 | 9</td><td>9 | 9</td><td>9 | 9</td><td>2 | 10</td><td>2 | 14</td><td>2 | 5</td><td>4 | 7</td><td>9 | 9</td><td>0 | 0</td><td>9 | 9</td><td>9 | 8</td><td>0 | 0</td><td>0 | 0</td><td>0 | 0</td><td>0 | 0</td><td>0 | 0</td><td>0 | 0</td></tr>
<tr><td>Total (max. 112)</td><td>42 | 41</td><td>42 | 42</td><td>38 | 37</td><td>38 | 37</td><td>21 | 32</td><td>27 | 44</td><td>26 | 29</td><td>28 | 29</td><td>41 | 42</td><td>35 | 31</td><td>43 | 43</td><td>45 | 43</td><td>23 | 23</td><td>22 | 22</td><td>23 | 23</td><td>21 | 22</td><td>30 | 31</td><td>30 | 31</td></tr>
<tr><td>Pin Prick Sensory Scores
L1-S2 dermatomes subscore (right | left)</td><td>0 | 1</td><td>1 | 4</td><td>0 | 0</td><td>0 | 0</td><td>0 | 13</td><td>3 | 17</td><td>0 | 0</td><td>0 | 0</td><td>0 | 0</td><td>0 | 0</td><td>9 | 0</td><td>9 | 0</td><td>0 | 0</td><td>0 | 0</td><td>0 | 0</td><td>0 | 0</td><td>0 | 0</td><td>0 | 0</td></tr>
<tr><td>Total (max. 56 | 56)</td><td>32 | 33</td><td>31| 35</td><td>17 | 16</td><td>15 | 15</td><td>27 | 41</td><td>36 | 50</td><td>13 | 15</td><td>15 | 13</td><td>32 | 28</td><td>30 | 31</td><td>43 | 34</td><td>44 | 34</td><td>22 | 22</td><td>21 | 22</td><td>24 | 24</td><td>21 | 21</td><td>29 | 30</td><td>30 | 30</td></tr>
</table>

Supplementary Table 1.
Neurological status of participants before and after EES^{REHAB}

exemplary factor loadings

	TEMPORAL GAIT FEATURES	PC1	PC2
1	Cycle duration (s)	-0.1576717	-0.0191449
2	Cycle velocity (cm/s)	0.71765717	0.29440406
3	Stance duration (s)	0.33582429	-0.3705423
4	Swing duration (s)	-0.4934916	0.32363859
5	Relative stance duration (% of gait cycle)	0.71023334	-0.359373
6	Double stance duration (%)	0.60312357	-0.2502677

	LIMB TRAJECTORIES		
7	Stride Length (cm)	0.6316849	0.23280816
8	Step Length (cm)	0.5614388	0.34622112
9	3D toe path length (cm)	0.5916267	0.43111612
10	Maximal backward position of foot (cm)	-0.5463889	0.59951176
11	Maximal forward position of foot (cm)	-0.6926062	0.53389382
12	Step Height (normalised)	0.72236662	0.10545746
13	Step Height (cm)	0.69784106	0.20966774
14	Max speed during swing (cm/s)	0.89591401	0.21245806
15	Time of max velocity during swing (% duration)	-0.1168049	0.07212136
16	Acceleration at swing onset (cm/s^2)	0.78410584	0.26677412
17	Endpoint velocity (cm/s)	0.62851802	0.15385477
18	Velocity vector at swing onset (deg)	-0.0894592	0.08398648

	DRAG		
19	Drag duration (s)	-0.5417939	0.3603316
20	Drag duration (%)	-0.8373842	0.17578048

	STABILITY			
21	Lateral displacement during swing (cm	Base of Support	0.05691657	0.00178616
22	Stance width (cm)		0.4236455	-0.2945496
23	Pelvis max vertical movement		0.32070531	0.34089065
24	Pelvis min vertical movement		-0.146866	0.25713701
25	Amplitude of pelvis vertical movement		0.6460952	0.16661574
26	Variability of saggital trunk oscillation	Trunk Stability and Oscillations	0.53231482	0.01633459
27	Velocity of saggital trunk oscillation		0.76830774	0.27624926
28	Variability of vertical hip oscillation		0.66372851	0.06598395
29	Variability of medio-lat hip oscillation		0.58622279	0.11184576
30	Variability of hip rotations		0.6548397	0.17146907

exemplary factor loadings

	JOINT ANGLES AND SEGMENTAL OSCILLATIONS		PC1	PC2
31	Crest oscillations (deg)	Backward	0.00455681	-0.1268226
32	Thigh oscillations (deg)		0.17662466	-0.5597325
33	Shank oscillations (deg)		-0.3169744	-0.0503729
34	Foot oscillations (deg)		0.23729717	-0.6326635
35	Whole limb oscillations (deg)		-0.1183546	-0.4490518
36	Crest oscillations (deg)	Forward	0.39236189	-0.0480982
37	Thigh oscillations (deg)		0.65639533	-0.46911
38	Shank oscillations (deg)		0.46698448	-0.2054473
39	Foot oscillations (deg)		0.68490153	-0.5701471
40	Whole limb oscillations (deg)		0.71750685	-0.5190953
41	Hip Joint (deg)	Extension	-0.1734601	0.59996864
42	Knee Joint (deg)		-0.0337904	0.34799307
43	Ankle joint (deg)		-0.2004252	0.63830011
44	Whole lImb abduction (deg)	Abduction	0.03433273	0.28954361
45	Foot abduction (deg)		0.08665734	0.39377111
46	Hip Joint (deg)	Flexion	-0.5538434	0.39348434
47	Knee Joint (deg)		-0.5989274	0.34107686
48	Ankle joint (deg		-0.6415763	0.473096
49	Whole lImb adduction (deg)	Adduction	-0.2250942	0.13517821
50	Foot adduction (deg)		-0.1538514	0.0444364
51	Crest oscillations (deg)	Oscillation Amplitudes	0.59139626	0.0952798
52	Thigh oscillations (deg)		0.73214101	0.02554458
53	Shank oscillations (deg)		0.7759825	-0.1850331
54	Foot oscillations (deg)		0.68378825	-0.1976867
55	Whole limb oscillations (deg)		0.79581363	-0.2813106
56	Hip Joint (deg)		0.54185671	0.40830478
57	Knee Joint (deg)		0.64190896	0.03090613
58	Ankle joint (deg)		0.64712855	0.08329771
59	Whole lImb medio-lat oscillation (deg)		0.5193659	0.28178655
60	Foot medio-lat oscillation (deg)		0.1960371	0.26750767

exemplary factor loadings

	VELOCITIES		PC1	PC2
61	Whole limb oscillation velocity (deg/s)	Minimum angle velocities	-0.8464141	-0.18285
62	Hip oscillation velocity (deg/s)		-0.759858	-0.3463828
63	Knee oscillation velocity (deg/s)		-0.8054906	-0.3284002
64	Ankle oscillation velocity (deg/s)		-0.6920943	-0.1568301
65	Whole limb oscillation velocity (deg/s)	Maximum Angle velocities	0.90190547	0.04548126
66	Hip oscillation velocity (deg/s)		0.76680173	0.39026841
67	Knee oscillation velocity (deg/s)		0.8515911	0.14651785
68	Ankle oscillation velocity (deg/s)		0.81309484	0.25109988
69	Whole limb oscillation velocity (deg/s)	Amplitude of angle velocities	0.91926192	0.09321898
70	Hip oscillation velocity (deg/s)		0.79560219	0.37980295
71	Knee oscillation velocity (deg/s)		0.8825796	0.25975487
72	Ankle oscillation velocity (deg/s)		0.76572477	0.19610856

	INTRALIMB TEMPORAL COUPLING			
73	Correlation between pelvis and thigh	proximal distal	0.24428247	-0.3228218
74	Correlation between thigh and shank		0.1160031	-0.4449952
75	Correlation between shank and foot		0.00752821	-0.5059836
76	Correlation between hip and knee	proximal distal	-0.0175302	-0.1634833
77	Correlation between knee and ankle		-0.0274252	-0.1351518
78	Correlation between ankle and foot		0.06456071	-0.14037

	ROBOTIC SUPPORT FEATURES		
79	Body Weight Support (%)	-0.8008253	-0.112497
80	Horizontal Support Force (%)	-0.7955145	-0.0546379

Supplementary Table 2.

Gait parameters calculated from kinematic recordings during walking in mice

Group	n	Condition	Had SCI?	Had EES^{REHAB}?	Terminal condition	Therapeutic feature modelled
1	3	Uninjured	No	No	None	Uninjured
2	3	SCI	Yes	No	None	SCI
3	3	EES^{REHAB}	Yes	Yes	None	EES^{REHAB}
4	3	$SCI^{\rightarrow EES::walking}$	Yes	No	EES^{ON} for 30 minutes while walking	Walking with EES after SCI
5	3	$EES^{\mathit{REHAB}\rightarrow EES::walking}$	Yes	Yes	EES^{ON} for 30 minutes while walking	Walking with EES after EES^{REHAB}
6	3	$EES^{\mathit{REHAB}\rightarrow EES}$	Yes	Yes	EES^{ON} for 30 minutes (no walking)	Immediate effect of EES in the absence of walking after EES^{REHAB}
7	3	$EES^{\mathit{REHAB}\rightarrow cortex}$	Yes	Yes	Optogenetic stimulation of the motor cortex for 30 minutes to model voluntary control (no walking)	Immediate effect of residual descending input in the absence of EES after EES^{REHAB}
8	3	$EES^{\mathit{REHAB}\rightarrow EES::cortex::walking}$	Yes	Yes	Optogenetic stimulation of the motor cortex and EES^{ON} for 30 minutes while walking	Walking with residual descending input and EES after EES^{REHAB}

Supplementary Table 3.
Description of experimental conditions
