# Supplementary material for: The neurons that restore walking after paralysis
Source: Nature. 2022 Nov 9;611(7936):540–7. doi: 10.1038/s41586-022-05385-7 (PMC9668750; doi:10.1038/s41586-022-05385-7)
Supplement: Supplementary file 2 — Reporting Summary [file 41586_2022_5385_MOESM2_ESM.pdf]

nature portfolio

Corresponding author(s): Gregoire Courtine

Last updated by author(s): May 11, 2022

Reporting Summary

Nature Portfolio wishes to improve the reproducibility of the work that we publish. This form provides structure for consistency and transparency in reporting. For further information on Nature Portfolio policies, see our Editorial Policies and the Editorial Policy Checklist.

Statistics

For all statistical analyses, confirm that the following items are present in the figure legend, table legend, main text, or Methods section.

n/a	Confirmed	
☐	☒	The exact sample size (*n*) for each experimental group/condition, given as a discrete number and unit of measurement
☐	☒	A statement on whether measurements were taken from distinct samples or whether the same sample was measured repeatedly
☐	☒	The statistical test(s) used AND whether they are one- or two-sided<br>*Only common tests should be described solely by name; describe more complex techniques in the Methods section.*
☐	☒	A description of all covariates tested
☐	☒	A description of any assumptions or corrections, such as tests of normality and adjustment for multiple comparisons
☐	☒	A full description of the statistical parameters including central tendency (e.g. means) or other basic estimates (e.g. regression coefficient) AND variation (e.g. standard deviation) or associated estimates of uncertainty (e.g. confidence intervals)
☐	☒	For null hypothesis testing, the test statistic (e.g. *F*, *t*, *r*) with confidence intervals, effect sizes, degrees of freedom and *P* value noted<br>*Give P values as exact values whenever suitable.*
☒	☐	For Bayesian analysis, information on the choice of priors and Markov chain Monte Carlo settings
☒	☐	For hierarchical and complex designs, identification of the appropriate level for tests and full reporting of outcomes
☐	☒	Estimates of effect sizes (e.g. Cohen's *d*, Pearson's *r*), indicating how they were calculated

Our web collection on statistics for biologists contains articles on many of the points above.

Software and code

Policy information about availability of computer code

Data collection	Human Data: Muscular recordings were acquired with Delsys Trigno Plugin v2.0.2 integrated in Nexus v1.8.5;<br>Mouse Data: Labchart v8 (ADInstruments), Labview (2018 version 18.0), Zen2 Black (v2.3 Zeiss), Imaris (9.1.2, 64Bit, Bitplane).
Data analysis	All softwares and software versions used to analyze data are described in the Method section at the relevant paragraph. Following is a list of softwares used: Matlab v2020a, Imaris (Bitplane, v.9.0.0), ImageJ NIH (v2.0), Labchart v.8 (ADInstruments), Sim4Life by ZMT(v.4.5.0), Matrox Solios eCL-B, Arivis AG (v. 3.6.2), Munich, Germany, Neurolucida MBF Bioscience (v 11.03). Illustrations were generated using Autodesk 2020.2, Maya 2020.2, Adobe Illustrator CC 2015. R (version 26.2.0), Adobe Photoshop (version 23.3.2), PRISM version 8.3 (Graph Pad). Single-nucleus sequencing and spatial transcriptomics analysis was performed in R (version 3.6.0), using the following packages: Cell Ranger (version 2.1), Libra (version 1.0.0), Matrix (version 1.3-4), Matrix.utils (version 0.9.8), R.utils (version 2.11.0), RANN (version 2.6.1), RCTD (version 1.1.0), RNiftyReg (version 2.7.0), Seurat (version 4.0.5), argparse (version 2.1.3), broom (version 0.7.10), clustree (version 0.4.4), colorspace (version 2.0-2), data.table (version 1.14.2), drlib (version 0.1.1), emmeans (version 1.7.2), fgsea (version 1.20.0), ggdendro (version 0.1.22), ggplot2 (version 3.3.5), ggrastr (version 1.0.0), ggrepel (version 0.9.1), ggsci (version 2.9), ggstance (version 0.3.5), igraph (version 1.2.9), imager (version 0.42.13), lme4 (version 1.1-27.1), lmerTest (version 3.1-3), magrittr (version 2.0.1), ontologyIndex (version 2.7), patchwork (version 1.1.1), readxl (version 1.3.1), scales (version 1.1.1), scater (version 1.14.6), sparseMatrixStats (version 1.5.3), tgp (version 2.4-18), tidyverse (version 1.3.1), ungeviz (version 0.1.0), velocyto.R (version 0.6), viridis (version 0.6.2), zoo (version 1.8-9), and a custom fork of the Splatter package that has been previously described, available from https://github.com/jordansquair/splatter_batch. Augur is available from GitHub (https://github.com/neurorestore/Augur). Magellan is available from GitHub (https://github.com/neurorestore/Magellan). Libra is available from GitHub (https://github.com/neurorestore/Libra).

For manuscripts utilizing custom algorithms or software that are central to the research but not yet described in published literature, software must be made available to editors and reviewers. We strongly encourage code deposition in a community repository (e.g. GitHub). See the Nature Portfolio guidelines for submitting code & software for further information.

Data

Policy information about availability of data

All manuscripts must include a data availability statement. This statement should provide the following information, where applicable:

- Accession codes, unique identifiers, or web links for publicly available datasets
- A description of any restrictions on data availability
- For clinical datasets or third party data, please ensure that the statement adheres to our policy

Raw sequencing data and count matrices have been deposited to the Gene Expression Omnibus (GSE184370), snRNA-seq, and GSE184369, spatial transcriptomics.

Field-specific reporting

Please select the one below that is the best fit for your research. If you are not sure, read the appropriate sections before making your selection.

☒ Life sciences ☐ Behavioural & social sciences ☐ Ecological, evolutionary & environmental sciences

For a reference copy of the document with all sections, see nature.com/documents/nr-reporting-summary-flat.pdf

Life sciences study design

All studies must disclose on these points even when the disclosure is negative.

Sample size	Sample sizes are estimated based on previous physiological studies using similar animal models. We report one of the first proof-of-concept results in nine patients who contributed to a First-in-Man study. No previous data existed to predetermine sample size. Previous studies employing spinal cord stimulation or novel implanted neurotechnologies (e.g. brain machine interface) in individuals with spinal cord injury reported their results in 1 to 4 participants.
Data exclusions	The exclusion criteria for data was established prior to the experiments: In mice, expression of viral vectors were confirmed post-mortem. If there was a lack of expression, the animal was excluded from behavioural analysis. No data were excluded in human clinical data sets.
Replication	All tested conditions were repeated across multiple trials (at least 10 trails per mouse during at least two independent testing sessions) and the results averaged to obtain a single-subject mean performance. Histological assessments were repeated in at least 5 animals showing consistent outcomes.
Randomization	Animals were randomly assigned to groups. Randomization for human participants was not sought in the present study. Each participant served as their own control (stimulation off vs. on conditions; evaluations at different points over time throughout the rehabilitation training period). During voluntary experiments, participants were asked to take small or large steps in a randomized order.
Blinding	For kinematic analysis blinding was not possible (and irrelevant, due to behavioral changes across conditions), however the investigator was blinded to group allocation during data collection (i.e. recordings) and video tracking is a highly automatized task. All histological analysis were blinded. For clinical studies, investigators were not blinded. Their expertize was required to optimize the intervention and to apply the intervention during evaluations. Furthermore, the effects of the intervention were obvious, acutely producing changes in the kinematics and muscle activities of the participants during walking.

Reporting for specific materials, systems and methods

We require information from authors about some types of materials, experimental systems and methods used in many studies. Here, indicate whether each material, system or method listed is relevant to your study. If you are not sure if a list item applies to your research, read the appropriate section before selecting a response.

Materials & experimental systems

n/a	Involved in the study	
☐	☒	Antibodies
☒	☐	Eukaryotic cell lines
☒	☐	Palaeontology and archaeology
☐	☒	Animals and other organisms
☐	☒	Human research participants
☐	☒	Clinical data
☒	☐	Dual use research of concern

Methods

n/a	Involved in the study	
☒	☐	ChIP-seq
☒	☐	Flow cytometry
☒	☐	MRI-based neuroimaging

Antibodies

Antibodies used	Primary antibodies were: rabbit anti-GFAP (1:500, Dako Z0334), cfos (1:2000 Synaptic Systems 226003), vGluT1 (1:1000, Synaptic

Antibodies used	Systems 135302). Secondary antibodies were all Alexa Fluor Conjugated from ThermoFisher Scientific, USA: donkey anti-rabbit Alexa Fluor® 647 (1:1000, A-31573), donkey anti-rat Alexa Fluor® 647 (1:1000, A-48272), donkey anti-goat Alexa Fluor® 647 (1:1000, A-21447).
Validation	We only used commercial antibodies. All of them were quality controlled and validated by the manufacturer. Validation statements can be found on the manufacturer's website.

Animals and other organisms

Policy information about studies involving animals; ARRIVE guidelines recommended for reporting animal research

Laboratory animals	Adult male or female C57BL/6 mice (15-35 g body weight, 12-30 weeks of age) or transgenic mice were used for all experiments. vGluT2Cre (Jackson Laboratory 016963), Ai65(RCFL-tdT) (Jackson Laboratory 021875), Parvalbumin (PV)Cre (Jackson Laboratory 017320), and AdvillinFlpO (gifted by Dr. Victoria Abraira) and Vsx2Cre (MMMRRC 36672, also called Chx10Cre) transgenic mouse strains were bred and maintained on a mixed genetic background (C57BL/6). Housing, surgery, behavioral experiments and euthanasia were all performed in compliance with the Swiss Veterinary Law guidelines. Mice were maintained in house under standard housing conditions (12-hour light/dark cycle) with 24 hour access to water and standard chow diet at temperature at 21 +/-1 ° C and relative humidity at 55 +/-5%. All procedures and surgeries were approved by the Veterinary Office of the Canton of Geneva.
Wild animals	No wild animals were used in this study.
Field-collected samples	No field-collected samples were used in this study.
Ethics oversight	All procedures and surgeries were approved by the Veterinary Office of the Canton of Geneva (Switzerland). Housing, surgery, behavioral experiments and euthanasia were all performed in compliance with the Swiss Veterinary Law guidelines.

Note that full information on the approval of the study protocol must also be provided in the manuscript.

Human research participants

Policy information about studies involving human research participants

Population characteristics	Nine individuals who had suffered a traumatic thoracic SCI participated in the study. Their neurological status was evaluated according to the International Standards for Neurological Classification of Spinal Cord Injury. Patients fulfilling all of the following inclusion criteria may be enrolled in the study:<br>- Age 18-65 (women or men)<br>- Sensorimotor or motor complete and incomplete SCI graded as AIS A,B, C & D<br>- Level of lesion: T10 and above, based on AIS level determination by the PI, with preservation of conus function<br>- The intact distance between the cone and the lesion must be at least 60 mm.<br>- Focal spinal cord disorder caused by either trauma or epidural, subdural or intramedullary bleeding<br>- Minimum 12 months post-injury<br>- Completed in-patient rehabilitation program<br>- For ASIA C and D, able to stand with walker or 2 crutches<br>- Stable medical, physical and psychological condition as considered by Investigators<br>- Able to understand and interact with the study team in French or English<br>- Adequate care-giver support and access to appropriate medical care in patient's home community<br>- Agree to comply in good faith with all conditions of the study and to attend all required study training and visit<br>- Must participate in two training sessions before eligibility is confirmed<br>- Must provide and sign Informed Consent prior to any study related procedures<br><br>Exclusion Criteria. The presence of any one of the following exclusion criteria will lead to exclusion of the subject:<br>- Limitation of walking function based on accompanying (CNS) disorders (systemic malignant disorders, cardiovascular disorders restricting physical training, peripheral nerve disorders)<br>- History of significant autonomic dysreflexia<br>- Cognitive/brain damage<br>- Epilepsy<br>- Patient who has spinal canal stenosis<br>- Patient who uses an intrathecal Baclofen pump.<br>- Patient who has any active implanted cardiac device such as pacemaker or defibrillator.<br>- Patient who has any indication that would require diathermy.<br>- Patient who has any indication that would require MRI.<br>- Patients that have an increased risk for defibrillation<br>- Severe joint contractures disabling or restricting lower limb movements.<br>- Haematological disorders with increased risk for surgical interventions (increased risk of haemorrhagic events).<br>- Participation in another locomotor training study.<br>- Congenital or acquired lower limb abnormalities (affection of joints and bone).<br>- Women who are pregnant (pregnancy test obligatory for woman of childbearing potential) or breast feeding or not willing to take contraception.<br>- Known or suspected non-compliance, drug or alcohol abuse.<br>- Spinal cord lesion due to either a neurodegenerative disease or a tumour.<br>- Patient has other anatomic or co-morbid conditions that, in the investigator's opinion, could limit the patient's ability to participate in the study or to comply with follow-up requirements, or impact the scientific soundness of the study results.<br>- Patient is unlikely to survive the protocol follow-up period of 12 months.

March 2021

Recruitment

Participant recruitment was done via the clinicaltrial.gov website where the principal investigators' contact details were disclosed (NCT02936453). Patients and physicians contacted them directly to communicate their interest to participate or to refer a patient to the STIMO study. The clinical study nurse communicated with the patients or the referring physician and reviewed the clinical status of the patient for compliance with the inclusion and exclusion criteria listed below. Patients meeting the inclusion criteria were given the study's flyer and the informed consent form to understand further their implications and involvement within this clinical study. The participants' selection was also based on their ability to live independently and their autonomy in their daily living activities.
Regrading the recruitment biases, all the inclusion and exclusion criteria were strictly followed and were defined solely to ensure the proper conduct of the study. The patient enrolled had very diverse profiles and form a good representation of the SCI population able to follow intensive locomotor rehabilitation.

Ethics oversight

This study was approved by the Swiss ethical authorities (Swissethics protocol number 04/2014 ProjectID: PB_2016-00886, Swissmedic protocol 2016-MD-0002) and was conducted in accordance with the Declaration of Helsinki.

Note that full information on the approval of the study protocol must also be provided in the manuscript.

Clinical data

Policy information about clinical studies

All manuscripts should comply with the ICMJE guidelines for publication of clinical research and a completed CONSORT checklist must be included with all submissions.

Clinical trial registration

NCT02936453

Study protocol

https://clinicaltrials.gov/ct2/show/NCT02936453

Data collection

Data on the 9 study subjects was collected between May 2019 and May 2021 at Lausanne University Hospital (CHUV, Switzerland).

Outcomes

Primary endpoints
The overground, robot-assisted neurorehabilitation in combination with spinal EES will result in less assistance required to walk and faster speed of walking. This will be calculated within each individual and across the group (12 patients). Chosen measures: WISCI II Score, 10-Meter Walk Test, Weight Bearing Capacity (WBC).
Secondary endpoints
The overground, robot-assisted neurorehabilitation in combination with spinal EES will result in more independence in activities of daily living and an improved endurance during standing and walking. This will be calculated within each individual and across the group (12 patients). Chosen measures: SCIM III Score, 6-Minute Walk Test.
